# A Facial-Expression-Aware Edge AI System for Driver Safety Monitoring

**DOI:** 10.3390/s25216670

**Published:** 2025-11-01

**Authors:** Maram A. Almodhwahi, Bin Wang

**Affiliations:** Department of Computer Science and Engineering, Wright State University, Dayton, OH 45435, USA; almodhwahi.2@wright.edu

**Keywords:** driver monitoring system, convolutional neural networks, deep learning models, road safety, human emotions and activity recognition, human–machine interaction, in-vehicle monitoring, real-time monitoring

## Abstract

Road safety has emerged as a global issue, driven by the rapid rise in vehicle ownership and traffic congestion. Human error, like distraction, drowsiness, and panic, is the leading cause of road accidents. Conventional driver monitoring systems (DMSs) frequently fail to detect these emotional and cognitive states, limiting their potential to prevent accidents. To overcome these challenges, this work proposes a robust deep learning-based DMS framework capable of real-time detection and response to emotion-driven driver behaviors that pose safety risks. The proposed system employs convolutional neural networks (CNNs), specifically the Inception module and a Caffe-based ResNet-10 with a Single Shot Detector (SSD), to achieve efficient, accurate facial detection and classification. The DMS is trained on a comprehensive and diverse dataset from various public and private sources, ensuring robustness across a wide range of emotions and real-world driving scenarios. This approach enables the model to achieve an overall accuracy of 98.6%, an F1 score of 0.979, a precision of 0.980, and a recall of 0.979 across the four emotional states. Compared with existing techniques, the proposed model strikes an effective balance between computational efficiency and complexity, enabling the precise recognition of driving-relevant emotions, making it a practical and high-performing solution for real-world in-car driver monitoring systems.

## 1. Introduction

Road safety has been a global issue driven by the rapid increase in vehicle ownership and the expansion of transportation infrastructure [1]. As reported by Hedges et al. [2], the global vehicle fleet reached 1.47 billion in 2023, with projections of continued growth in the coming future. The World Health Organization (WHO) reports that road traffic accidents represent a global public health crisis, causing approximately 1.35 million fatalities annually, a figure that continues to increase [3]. Notably, road accidents rank as the eighth leading cause of death worldwide, accounting for 2.5% of all global mortality [3].

Koesdwiady et al. [4] state that 90% of road accidents are caused by human errors. The leading causes of human errors include fatigue, drowsiness, and distractions. The economic toll of these accidents is substantial, with traffic-related incidents costing nations an estimated 3–5% of their annual Gross Domestic Product (GDP).

WHO identifies five key risk factors for road accidents: speeding, drunk driving, non-use of motorcycle helmets, seat belt non-compliance, and lack of child restraint systems. While global initiatives have targeted driver negligence and distraction, progress remains insufficient. According to [3], 10 countries achieved a 50% reduction in road fatalities between 2020 and 2021, while another 35% saw declines of 39–40%. Though promising, these results emphasize the critical need for technological innovation. To sustain this progress, integrating advanced technologies that address root causes of accidents will be essential for further reducing global road fatalities.

Driver monitoring systems (DMSs) enhance driver safety by continuously tracking driver behavior, detecting signs of drowsiness, distraction, or unsafe practices, and providing real-time alerts to prevent accidents. Beyond immediate monitoring, DMS collects valuable behavioral data over time, enabling personalized feedback that helps drivers recognize and improve their driving patterns. This feedback loop encourages greater awareness and promotes safer driving habits. In addition, the collected data serves multiple stakeholders: fleet managers can optimize operations, insurance companies can refine risk assessments, and researchers can identify key risk factors for accidents. All of these collectively contribute to broader transportation safety improvements. The adoption of DMS is on the rise and is becoming a part of the growing Internet of Vehicles (IoV) industry.

Globally, the adoption of driver monitoring systems is on the rise and in high demand. According to a report by Grand View Research, the global market for driver monitoring systems (DMSs) was estimated to be worth USD 3.03 billion in 2024 and is expected to grow at a compound annual growth rate (CAGR) of 11.7% between 2025 and 2033, reaching USD 8.10 billion [5].

Despite their benefits, current DMSs face limitations in reliably detecting driver emotions and facial cues across diverse real-world conditions. Challenges arise from factors such as high-speed driving, poor lighting, rough or uneven road surfaces, adverse weather conditions (e.g., rain, snow, or wind blowing through open windows), sudden driver movements (e.g., abrupt head turns/distraction and drunk driving [6]), and camera malfunctions that degrade image quality. Nevertheless, DMSs remain a critical tool for road safety, as they effectively identify risky behaviors, improve driver self-awareness, and ultimately help reduce accidents and fatalities.

In this work, we introduce an intelligent driver monitoring system (DMS) that uses behavioral analysis and real-time facial expression analysis to improve road safety. We demonstrate the deployment of our CNN-based Inception model on the MAX78000FTHR microcontroller (Analog Devices Inc., Mansfield, TX, USA) where it delivers high-accuracy (98.6% overall, F1-score up to 1.0), real-time emotion detection by maintaining optimal processing efficiency.

Our system accurately detects fatigue, distraction, fear, and neutrality, the critical states that have a direct impact on driving safety, unlike traditional DMSs that focus solely on fatigue. The system delivers reliable nighttime driving monitoring, maintaining high performance despite partial face turns, low light, or erratic inputs like bumpy roads. Its training on diverse, multi-view datasets ensures robust generalizability across ages and cultural characteristics, making it highly suitable for real-world vehicle integration.

The remainder of this paper is organized as follows. Section 2 provides a comprehensive review of existing approaches to driver monitoring and facial expression recognition, along with related studies and commercial DMS products for facial expression monitoring, establishing the foundation for our contribution. Section 3 introduces the proposed system architecture, highlighting its components, data flow, and the set of driving-related emotions under consideration. Section 4 presents the learning model pipeline, including data collection, labeling, preprocessing, facial feature analysis, and classification strategies. Section 5 explains the methodology used for model training and testing, while Section 6 reports the performance evaluation outcomes. Section 7 places our model within the state of the art by comparing it against four recent and well-known driver emotion recognition frameworks. Section 8 discusses the hardware realization of the system, including microcontroller selection, multimodal sensor integration, configuration, system flow, and performance demonstration. Section 9 provides a comparative analysis with existing driver monitoring solutions, emphasizing the novelty and practicality of our approach. Finally, Section 10 concludes the paper by summarizing the principal findings and outlining potential directions for future research.

## 2. Related Work

### 2.1. Driver Safety Monitoring Approaches

A DMS is an IoT-based solution that tracks and analyzes driver behavior in real time. By leveraging sensors, cameras, and AI-powered algorithms, DMS continuously monitors driver actions, fatigue levels, and emotional states to improve road safety and driving performance. Specifically, it tracks and analyzes four key categories of data:

1. External Vehicle Environment: Road conditions, traffic, and surrounding obstacles;
2. Internal Vehicle Environment: Cabin conditions, passenger presence, and potential distractions;
3. Driver Behavior: Steering patterns, braking habits, and adherence to traffic rules;
4. Driver State: Fatigue levels, emotional responses, and attentiveness.

**Figure 1:**
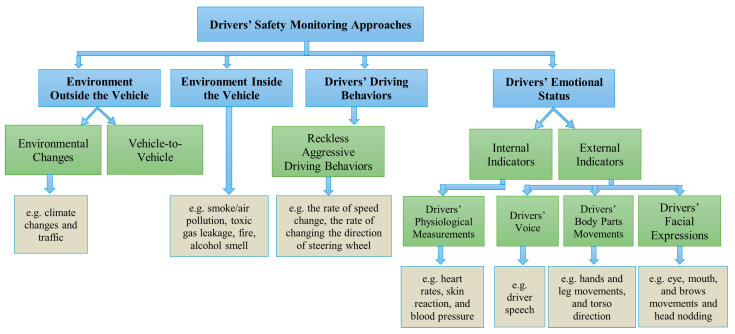
Driver safety monitoring approaches.

Each of these perspectives provides unique insights, contributing to a comprehensive understanding of driver safety. Existing driver safety monitoring approaches that address these perspectives are summarized in Figure 1.

Traditional driver behavior assessment relies on physiological measures such as heart rate, blood pressure, and Electroencephalogram (EEG) brain monitoring. Emotion detection uses both internal signals (EEG and Galvanic Skin Reaction (GSR)) and external cues (facial expressions, gestures, and speech). With technological advancements in vehicles such as artificial intelligence (AI) and machine learning (ML), modern DMSs can analyze changes in facial expressions, providing a proactive approach to accident prevention.

The driver’s facial expressions, particularly subtle movements of the mouth, eyes, and head, serve as critical indicators of cognitive and emotional state. For in-car systems, facial expression analysis proves to be most practical. While drivers typically maintain neutral states, their facial features change dynamically in response to traffic, road conditions, stress, and emotions, making facial recognition ideal for real-time driver state monitoring.

According to studies conducted on driver expressions using in-car facial recognition technology, facial expressions change many times per minute [7], especially when the driver is reacting to unexpected changes in road conditions. Facial expressions effectively detect emotions since the face is the most expressive and the immediate indicator of emotions [8]. Furthermore, facial expressions provide clues about the driver’s current status and therefore help alert the driver before the occurrence of a potentially dangerous situation.

### 2.2. Facial Expression Detection and Analysis

Previous work on the detection, analysis, and identification of driver facial expressions is summarized and compared in Table 1 which includes 13 studies published between 2018 and 2023. We exclude studies that examine the movements of the driver’s body parts, physiological parameters, driving behaviors and patterns, or the environment inside or outside the vehicle.

In order to interpret and understand emotional states that impact driving performance and safety, this study focuses on observing drivers’ facial expressions. Deep learning-based Facial Emotion Recognition (FER) systems have been the subject of numerous investigations, using various architectures, datasets, and optimization techniques to improve robustness in various scenarios. Using CNN and SVM classifiers in conjunction with conventional face detection techniques (Viola–Jones, MTCNN), early studies like Zhang et al. (2017) [12] achieved high accuracy (90.7%). Later models, such as those by Shang et al. (2023) [15], placed more emphasis on deep fusion networks and lightweight Xception variants to increase robustness to illumination and occlusion. Hybrid frameworks that integrate multiple modalities were introduced by more recent approaches. For instance, Sudha and Suganya (2023) [10] used CNN + SVM architectures to combine physiological, vocal, and facial features, and they were able to achieve up to 95.92% accuracy on datasets such as CK+, KDEF, FER2013, and KMU-FED. Similarly, Jain et al. (2023) [9] achieved 99.69% accuracy on the KDEF dataset by using Squirrel Search Optimization with GRU and RetinaNet.

Numerous studies tackled practical issues like dim lighting or hazy photos. To enhance performance on mobile and embedded systems, Sahoo et al. (2022) [14] and Shang et al. (2023) [15] created lightweight CNN models (such as SqueezeNet, SICNET+RC-NET, and RS-Xception). Nonetheless, the majority of models continue to exhibit decreased accuracy in low light levels. Hilal et al. (2022) [18] and Xiao et al. (2022) [19] investigated explainable AI and transfer learning approaches for incorporation into Advanced Driver Assistance Systems (ADASs). Their results demonstrate the balance between interpretability and computational efficiency. For Kolmogorov–Arnold Networks [22], they used a custom dataset created in collaboration with a public-transportation partner company where bus drivers’ actions like mobile phone usage and interaction with passengers were recorded and tested on the model, achieving 39.49–97.39% accuracy with the ability to work on dark and blurry images. The body of research shows that deep learning models can correctly identify emotions like anger, fear, happiness, sadness, and surprise, but that their performance suffers in dimly lit or obscured environments. For in-vehicle monitoring systems, these studies highlight the need for more varied datasets, reliable architectures, and real-time embedded implementations.

### 2.3. Existing DMS Products with Facial Expressions Monitoring

A comparison of the strengths of our proposed DMS with existing products is given in Table 2.

For real-time user interaction and virtual reality applications, Seeing Machines’ FaceAPI tracks eye position and facial movement. But it does not work well in different types of light. Next, for the Jungo-CoDriver System, it keeps track of passenger occupancy (e.g., seat belt use and unattended child), driver fatigue, and distraction, which makes it helpful for safety, but it frequently misinterprets actions. For the AI-based imaging engine for real-time image enhancement and facial analytics that is called Xperi-FotNation, its primary disadvantage is that it requires a lot of processing power, which restricts its use on low-power devices. Moving to Edge3 Technologies’ TRIFECTA, it uses AI trained on complex scenes to detect driver violations, microsleep, and fatigue. It is sensitive to changes in illumination, but has been tested in extremely hot and cold temperatures. The Denso’s Driver Status Monitor (DSM) uses near-infrared cameras to identify signs of inattention, negligence, and sleepiness at night. However, its indirect facial views reduce accuracy.

Next comes the Ellcie Healthy Virtual Lab. It uses smart eyewear that tracks physiological data to identify sleepiness and falls. It is restricted to eye movement analysis and is uncomfortable to wear for extended periods of time. For Optalert’s Smart DMS, it uses infrared sensors and the Johns Drowsiness Scale (JDS) to identify microsleep and drowsiness, but it is unable to evaluate more general emotional states. On the other hand, for the Gentex Full Display Mirror, it is an intelligent mirror with glare reduction and iris scanning that improves safety and visibility. It does not, however, keep an eye on the feelings or actions of drivers. Next is the Smart Eye Pro, which is a multi-camera system that tracks eyes in 360 degrees, but is not integrated with behavioral or emotional DMS features. The ZF-CoDRIVE, on the other hand, does not evaluate a driver’s emotions or level of alertness, but it does support situational awareness and semi-autonomous driving. Next is the OmniVision OV2311 Sensor (OmniVision Technologies, Inc., Santa Clara, CA, USA) which is an inexpensive NIR image sensor that can identify alertness and eye movement but not emotions. Finally, we can see that the camera-based DMS from Seeing Machines & Autoliv tracks the head, face, and eyelids in real time to detect distractions and drowsiness, but it has trouble with hazy or blurry images.

In summary, as we have reviewed above, many existing DMSs leverage facial expression analysis to enhance driving safety and experience. However, these systems face several limitations, including high cost, implementation complexity, and reduced effectiveness in challenging environments, such as low-light conditions, uneven roads, or low-resolution imaging. To address these issues, this work focuses on improving DMS accuracy, efficiency, and portability, ensuring robust performance even in non-ideal scenarios. We present an enhanced DMS that leverages state-of-the-art techniques to predict and evaluate risky driving behaviors through real-time analysis of the driver’s emotional state as captured by facial expression recognition.

## 3. System Overview

**Figure 2:**
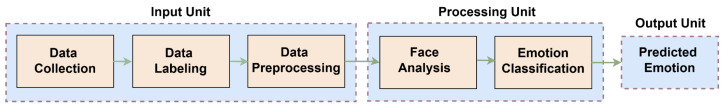
System overview.

Our system focuses on monitoring drivers’ adequacy for driving by capturing and analyzing their facial expressions to understand what emotion they convey. The system detects and classifies emotional states in real time, enabling the identification of potential safety risks associated with impaired driving capacity. The system operates a multi-stage pipeline consisting of (1) data acquisition and preprocessing, (2) model training and validation, and (3) risk assessment output generation. Figure 2 presents the complete system architecture.

The system consists of the following components: (1) input unit, (2) processing unit, and (3) output unit.

### 3.1. Input Unit

**Figure 3:**
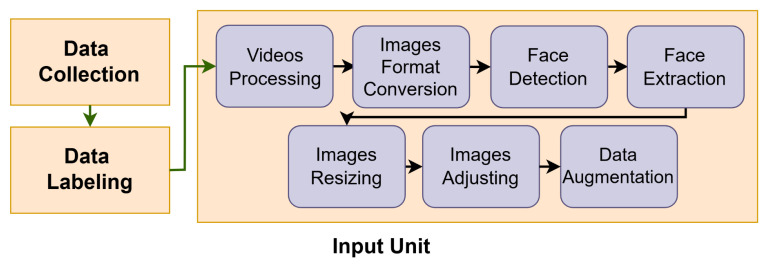
The input unit.

This unit consists of data collection, data labeling, and data preprocessing. In the data collection section, we provide a detailed description of the data collection process, the datasets employed, and their defining characteristics. Next, in the data labeling section, we go into details about the characteristics of each emotion and the data labeling steps. Lastly, in the data processing section, we discuss the processing steps in detail, including video processing, image format conversion, face detection, face extraction, data augmentation, image resizing, and image adjusting. Figure 3 presents the components of the input unit.

### 3.2. Processing Unit

**Figure 4:**
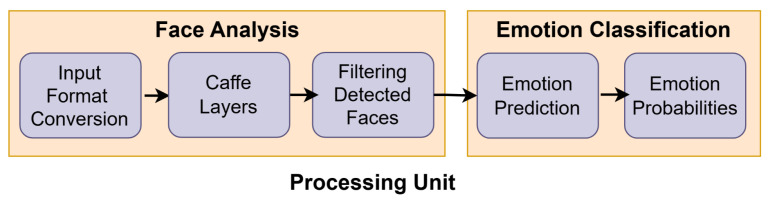
The processing unit.

This unit consists of three main components: face analysis, emotion extraction, and classification. Once the facial data are fed into the model, they are analyzed accordingly by a CNN-based method to automatically learn features of different alarming facial expressions (distraction, drowsiness, and fear), as well as the neutral face expression. Figure 4 presents the components of the processing unit.

### 3.3. Output Unit

In this step, the system generates a vector of probabilities corresponding to each class of facial expressions. The classes represent the following emotions: distraction, drowsiness, fear, and neutrality. The emotional state corresponding to the highest probability is considered the dominant emotion.

### 3.4. System Dataflow

**Figure 5:**
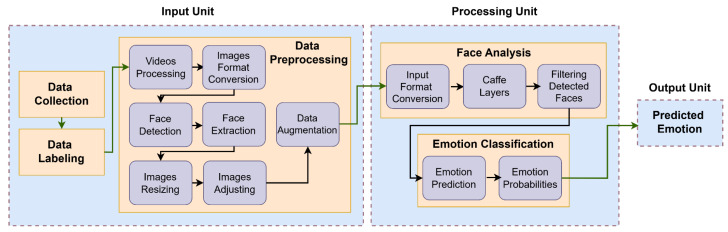
System data flow.

Figure 5 illustrates the data flow of the system. Specifically, we have the following:

■ Data Collection: Images and videos are obtained from different datasets and loaded by the system.
■ Data Labeling: Images and video frames collected from various datasets are manually labeled and sorted into four separate subfolders to ensure proper categorization.
■ Data Preprocessing: Raw images and video frames undergo preprocessing to prepare them for input into the system and to ensure compatibility with the system’s input requirements.
■ Face Detection and Extraction: During the preprocessing stage, faces are detected using the DNN-based method, and the corresponding facial regions are extracted to produce images containing human faces only.
■ Face Analysis: The extracted facial data are fed into the model, where they are analyzed by a CNN-based method to automatically learn the distinctive features of various driver facial expressions (distraction, drowsiness, and fear), as well as the neutral face expression.
■ Emotion Prediction: The model computes and outputs the probability distribution across all facial expression categories.
■ Output Interpretation: The output probability distribution is examined to determine the probability of the maximum value, which is then mapped to the corresponding facial expression label (e.g., ’Neutral’ and ’Drowsy’). The system subsequently displays the emotion with the highest predicted probability.

### 3.5. Emotions Detected by the Proposed System

Drowsiness reflects fatigue and reduced vigilance. The model relies on eye closure sequences and reduced motion energy to detect it. On the other hand, distraction involves an attention shift. The DMS typically detects it through gaze deviation and lack of frontal face alignment, while the increased alertness brought on by fear results in tense facial muscles and widened eyes. In a DMS, this appears as an increased eye aspect ratio, and brief but intense gaze movements. Finally, neutrality serves as the baseline state for classification. It enables the model to measure deviation toward stress (fear), inattention (distraction), or fatigue (drowsiness).

Our DMS uses a combination of facial muscle dynamics, eye mouth activity, and head pose variations to distinguish between the four emotional states: fatigue/drowsiness, distraction, fear/panic, and neutrality. Table 3 below makes clear the characteristics of the driver’s face that can be used to measure the distinct facial movement patterns and temporal changes brought on by each emotion.

These distinctive features enable the DMS model to learn and classify emotions.

The emotions described above are among the most commonly experienced by drivers while on the road [36]. A study of commercial drivers found that 75% reported committing at least one driving error due to fatigue [37]. Research shows that anxiety (panic or fear) affects 40 million U.S. adults annually. In fact, 66% of Americans report experiencing driving anxiety, with 55% experiencing it during routine driving maneuvers [38]. According to an Allianz study [39], smartphone-related distractions while driving have risen sharply, with the percentage of drivers texting behind the wheel surging from 15% in 2016 to 24% in 2022.

These facial expressions serve as key danger indicators for driver states. The facial features and their corresponding emotions detected, such as widened eyes (alertness or stress), drooping eyelids (fatigue), and frequent glances away from the road (distraction), directly reflect impaired driving conditions [40]. Such manifestations of heightened stress or distraction can significantly compromise driving performance and overall safety.

## 4. The Learning Model

The architecture of the learning model and its processing stages are explained in detail in this section.

### 4.1. Data Collection

Using a representative dataset is essential for developing an effective deep learning (DL) system. Although many datasets have been published, the existing literature still lacks a standardized dataset covering most traffic classifications.

There are many challenges associated with dataset standardization, starting from the existing dataset being of limited scope, having biased composition, and being imbalanced in classes to having emotion labeling variability across different datasets and being real-world representative.

We curate and construct the dataset for our work from various data sources from datasets in Table 4 to ensure diversity. Portions of the research in this paper use the FERET database of facial images collected under the Facial Recognition Technology (FERET) program, sponsored by the Counterdrug Technology Development Program Office (DOD).

### 4.2. Data Labeling

Labeling facial features for expression recognition can be highly complex due to the many facial components involved in analysis. Previous studies have employed varying combinations of facial features to detect emotions, along with different emotion-specific characteristics. However, in our work, we focus on the eyes and mouth, key facial features that humans naturally prioritize when interpreting expressions. These regions convey a wide range of emotions and play a critical role in emotion recognition, along with head orientation. Since precise emotion recognition depends on distinguishing unique expressive patterns to avoid classification ambiguity, we carefully perform data labeling. Specifically, (1) distraction is characterized with open eyes, a closed mouth, and a tilted or rotated head, (2) drowsiness is characterized with closed or semi-closed eyes, a closed or open mouth, and a tilted or forward-facing head, (3) fear is characterized with wide-open eyes, a wide-open mouth, and a forward-facing head, and (4) neutrality is characterized with open eyes, a closed mouth, and a balanced forward-facing head.

We label the raw data obtained from different sources to create our dataset. The raw data are organized into 4 subfolders, each representing an emotion (e.g., fear, distraction, drowsiness, and neutrality) and containing images and videos of that emotion. During the training process, TensorFlow methods read the images, assign them labels based on the subfolder names, and map the subfolder names to numeric values, with each numeric value representing an emotion.

### 4.3. Data Preprocessing

**Figure 6:**
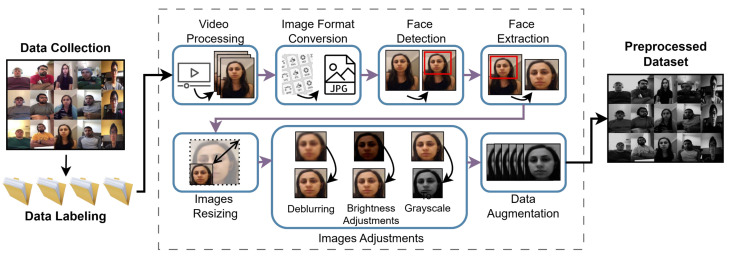
Data preprocessing.

Preprocessing is critical for ensuring that the model receives the input in a consistent format and can learn relevant features without being biased or sensitive to unnecessary variations in the data. All data preprocessing steps are illustrated in Figure 6.

Video Processing

We use the VLC Media Player to extract video frames and save them as JPEG images. These frames are then stored in a shared folder alongside other dataset images to expand the collection. To ensure standardization, we remove any frames with distortions, such as background noise or blocking.

Image Format Conversion

We standardize image formats by converting them into .jpg format using Python’s Pillow library. This results in a dataset of uniformly formatted images, though their dimensions may vary. The conversion to .jpg ensures consistency in compression, color channels, and bit depth, which improves processing efficiency and maintains image quality for downstream tasks.

Face Detection

For face detection, we employ OpenCV’s Deep Neural Network (DNN) module to create a Convolutional Architecture for Fast Feature Embedding model (Caffe model). Using our dataset, the model detects, localizes, and extracts human faces from the dataset images efficiently. We select a DNN-based framework for face detection due to its robustness in handling diverse variations, including differences in angle, scale, orientation, lighting, and pose. The framework’s automatic feature extraction capability eliminates the need for manual feature engineering, improving efficiency. Additionally, scaling the model enhances both robustness and accuracy. Its high adaptability also enables seamless integration with other tasks, facilitating future enhancements.

Caffe utilizes a layered architecture of interconnected neurons, where sequential data processing enables face detection. Our implementation combines ResNet-10 and SSD (Single Shot MultiBox Detector) models. ResNet-10 provides an optimal balance of accuracy and computational efficiency through its 10-layer residual network, while SSD enhances detection speed through its single-pass deep learning approach.

The Caffe framework processes data through multiple layer types, including the following:

■ Input layer;
■ Feature extraction layers (convolutional, pooling, Rectified Linear Unit (ReLU));
■ Normalization layers (batch normalization, dropout);
■ Output layers (softmax, loss);
■ A final concatenation layer for feature integration.

This hybrid architecture leverages the strengths of both ResNet-10 and SSD to achieve robust face detection with optimal speed–accuracy trade-offs.

Our Caffe-based model begins by defining the input and output directories for image loading. After loading the images, they are converted into the “blob” format required by Caffe. The neural network then processes these blobs to perform face detection and filters out low-confidence detections, applying a confidence threshold of 0.7. This step ensures that only the most reliable detections are kept. Finally, the model outputs a list of bounding boxes, each representing the coordinates of a detected face.

**Figure 7:**
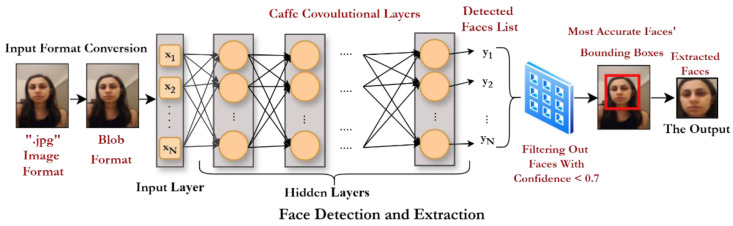
Face detection and extraction.

Figure 7 below illustrates the face detection process.

Face Extraction

Once the bounding boxes are generated, the code extracts the face regions from the images using their respective coordinates. This step involves cropping each detected face while discarding the surrounding background, ensuring only the relevant facial features are retained. The extracted faces are then saved into their corresponding class folders to preserve label accuracy.

Image Resizing

After face extraction, images are resized to 240 × 240 pixels. For variable-sized inputs, we preserve the aspect ratio using the following equations:

(1) $$\begin{array}{c} \mathrm{original}\_\mathrm{image}\_\mathrm{size}=(\mathrm{height},\mathrm{width}) \end{array}$$

(2) $$\begin{array}{c} \mathrm{aspect}\_\mathrm{ratio}=\mathrm{minimum}(\frac{\mathrm{targeted}\_\mathrm{height}}{\mathrm{original}\_\mathrm{height}},\frac{\mathrm{targeted}\_\mathrm{width}}{\mathrm{original}\_\mathrm{width}}) \end{array}$$

(3) $$\begin{array}{c} \mathrm{new}\_\mathrm{image}\_\mathrm{size}=(\mathrm{int}(\mathrm{original}\_\mathrm{width}\times \mathrm{aspect}\_\mathrm{ratio}),\mathrm{int}(\mathrm{original}\_\mathrm{width}\times \mathrm{aspect}\_\mathrm{ratio}) \end{array}$$

(4) $$\begin{array}{c} \mathrm{new}\_\mathrm{resized}\_\mathrm{image}=\mathrm{resize}(\mathrm{image},\;\mathrm{new}\_\mathrm{width},\mathrm{new}\_\mathrm{height}) \end{array}$$

To maintain aspect ratios during resizing, we calculate a scaling factor based on the original image dimensions and target size (240 × 240 pixels). Using this factor, we derive the new dimensions and perform the resizing accordingly. The equations above ensure proportional scaling throughout this process.

Image Adjusting

After the DNN generates the face dataset, we resume processing by converting all images to 8-bit unsigned integer format (uint8). This conversion ensures compatibility with OpenCV’s processing requirements. The pipeline then verifies each image’s color format by examining its channel count. Images with three channels (BGR format) are converted to grayscale, reducing computational complexity while preserving relevant features for facial expression analysis. This dimensionality reduction is particularly effective, as color information contributes minimally to expression recognition tasks.

Next, the images then undergo binarization using a fixed threshold of 200 and converting the pixel values into two levels: black and white. Pixel intensities below this threshold are set to 0 (pure black), while values above are set to 255 (pure white). This binary transformation offers four key advantages: (1) data simplification for more efficient processing, (2) improved edge and feature detection, enhancing contrast and simplifying feature extraction, (3) inherent noise reduction in the resulting image, and (4) reduced illumination effects that may occur due to varying lighting conditions inside the vehicle.

After the previous steps, we assess image quality through three metrics: blurriness, brightness, and contrast. For blur detection, we compute the Laplacian variance of the grayscale image—a well-established focus metric in computer vision. The Laplacian and the variance Var(ΔI) of the Laplacian are computed using the equation

$$\Delta I=\frac{\partial^{2}I}{\partial x^{2}}+\frac{\partial^{2}I}{\partial y^{2}}$$

The algorithm calculates the variance of the Laplacian-transformed image, where lower variance indicates greater blurriness. When the variance falls below the defined threshold, which is 250 (indicating excessive blur), we apply a sharpening convolution kernel to enhance edge definition. This operation improves feature visibility while reducing image fuzziness.

The pipeline then evaluates illumination conditions by calculating the mean pixel intensity of the grayscale image. This quantitative brightness assessment compares the measured value against an optimal brightness threshold. When underexposure is detected (i.e., mean intensity below threshold), the system (1) computes a brightness correction factor as the ratio between target and current intensity and (2) applies this factor uniformly to all pixels through scalar multiplication, effectively normalizing the image brightness while preserving relative contrast relationships.

We assess image contrast by computing the standard deviation of pixel intensities—a direct measure of image contrast. When the calculated contrast exceeds optimal thresholds (indicating excessive vibrancy), the system (1) derives a normalization factor from the ratio between target and current contrast values and then (2) applies this factor through linear scaling to achieve balanced contrast levels.

For model compatibility, we finalize preprocessing by (1) expanding image dimensions to include batch size notation and (2) adding a third channel when required, ensuring proper tensor formatting for DNN/CNN architectures.

Data Augmentation

To enhance model robustness and generate additional training data, we employ several augmentation techniques. First, pixel values are normalized to the range [0, 1]. We then apply a series of transformations to simulate real-world variations: (1) shearing (up to 20% horizontally) to mimic different viewing angles, (2) random zooming (up to 20%) to improve scale invariance, (3) horizontal flipping to leverage directional symmetry in the dataset, and (4) rotation (up to 20 degrees) and random shifts (20% vertically/horizontally) to account for positional variability. These augmentations significantly improve the model’s generalization capability and overall performance.

Augmented Dataset

The preprocessed images, which are converted to grayscale, resized, and possibly enhanced through deblurring and contrast adjustment, are then normalized and prepared for batch processing. The final dataset is organized into four emotion-specific folders (i.e., neutrality, fear, distraction, and drowsiness), each containing approximately 10,000 images. Within each emotion category, the images are distributed as 60% normal lighting, 20% dim lighting, and 20% shaky/blurry (to simulate real-world variability).

The dataset is further split into 80% training and 20% testing subsets, with dedicated subfolders for each. This structured partitioning ensures robust model evaluation under diverse conditions. Once the dataset is prepared, the system proceeds to perform facial analysis to classify emotions.

### 4.4. Facial Analysis

The system is trained to analyze facial features, such as the eyes, mouth, and head, using preprocessed datasets. The primary objective is to extract meaningful features and generate a numerical representation suitable for classification. We employ a convolutional neural network for face analysis, leveraging its superior image processing capabilities and its ability to automatically learn discriminative features from raw images.

To enhance the performance and efficiency of the proposed method, we develop an Inception-based CNN model. The Inception architecture is selected for face analysis and feature extraction due to its ability to capture both fine-grained and dominant facial features while maintaining computational efficiency. Despite newer alternatives, the Inception model remains highly effective, offering superior speed, accuracy, and deployability, particularly in real-time, edge, and embedded systems.

The model includes two key functions: the Inception Module, serving as the foundational building block, and the Mini-Inception function, which iteratively applies the Inception Module to extract multi-scale features at different network depths. The Mini-Inception function aggregates these hierarchical features through concatenation, enabling comprehensive representation learning.

The Inception module consists of four parallel processing paths:

■ Dimensionality Reduction Path: A 1 × 1 convolution (64 filters) that reduces input depth while preserving essential information by converting RGB images to grayscale when needed.
■ Local Feature Extraction Path: A 1 × 1 convolution (96 filters) followed by a 3 × 3 convolution (64 filters) for dimensionality reduction and fine detail capture such as eye corners, wrinkles, or subtle mouth deformations.
■ Global Feature Extraction Path: A 1 × 1 convolution (16 filters) followed by a 5 × 5 convolution (64 filters) to capture broader spatial patterns such as overall facial regions and broader emotional patterns.
■ Pooling Path: 3 × 3 max pooling followed by 1 × 1 convolution (64 filters) to extract dominant features of the face, like the eyes and the mouth, while maintaining dimensionality.

**Figure 8:**
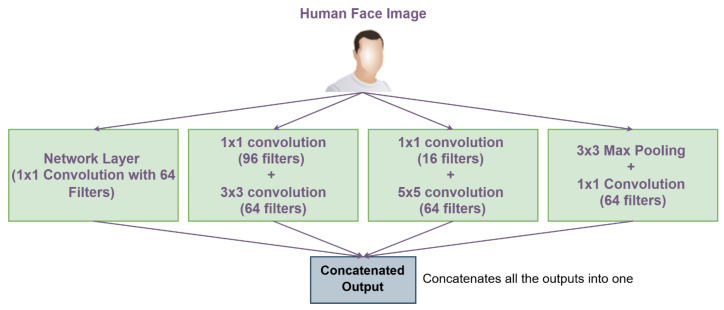
The inception model function.

The module concatenates outputs from all paths, combining multi-scale features into a unified representation, allowing the network to understand both local and global facial variations, which is an essential capability for distinguishing between similar emotions like neutrality and drowsiness. Figure 8 illustrates the inception model function.

The mini-inception function begins by processing the input image through the Inception module. The pipeline then executes the following sequence of operations:

■ Feature Extraction: A 3 × 3 separable convolution (16 filters) extracts spatial features.
■ Normalization: Batch normalization stabilizes and accelerates training.
■ Regularization: Dropout (25%) is applied to prevent overfitting.
■ Dimensionality Reduction: 2 × 2 max pooling decreases feature map dimensions.
■ This structure repeats iteratively, with each block increasing the filter count while maintaining the same operation sequence (Inception module → separable convolution → batch normalization → dropout → max pooling).

**Figure 9:**
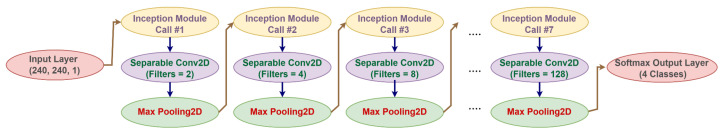
The Mini-Inception function.

The final layers consist of (1) a global pooling layer to reduce dimensionality and further prevent overfitting; (2) a Conv2D layer (4 filters) corresponding to the emotion classes; and (3) a softmax activation layer that generates class probabilities. The probability computation mechanism through the softmax layer is detailed in the subsequent Section 4.5. Figure 9 illustrates the Mini-Inception function.

### 4.5. Classification

This is the final stage of the system. As discussed in Section 4.4 CNNs excel at automatically learning features from images through successive layers. The output layer employs a softmax activation function, which converts the network’s raw logits into probabilities. The number of neurons in this layer corresponds to the four emotion classes, calculated using the following equation:

(5) $$\begin{array}{c} softmax\left(z_{i}\right)=\frac{e^{z_{i}}}{\sum_{j=1}^{n}e^{z_{j}}}. \end{array}$$

**Figure 10:**
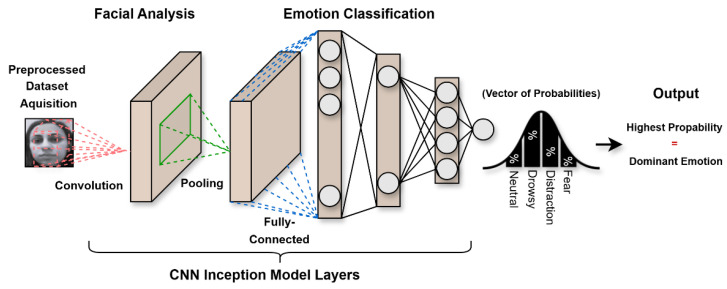
Facial analysis and classification.

The softmax function converts the logits into positive values by exponentiating them ($e^{z_{i}}$). These values are then normalized by dividing each exponent by the sum of all exponentials ($\sum_{j=1}^{n}e^{z_{j}}$), producing a probability distribution. The resulting vector represents the model’s confidence scores for each emotion class. Finally, the emotion with the highest probability is selected as the predicted (dominant) emotion. Figure 10 shows the facial analysis and classification process.

During training, we employ categorical cross-entropy as the loss function to quantify the discrepancy between the true emotion labels and the predicted probability distribution generated by the softmax function.

**Figure 11:**
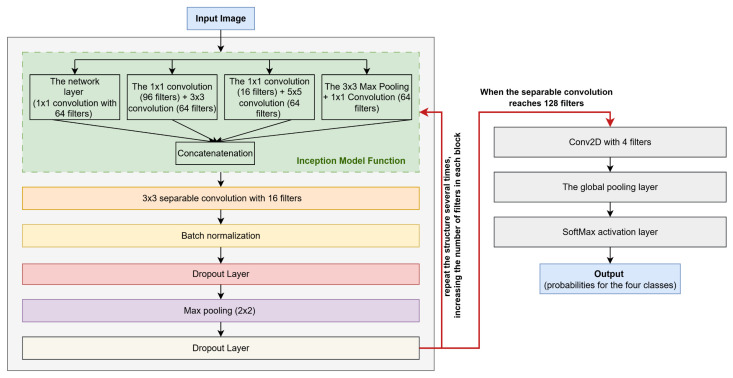
Deep learning network flow diagram.

If the model outputs equal probabilities for multiple emotions, a rare occurrence, the softmax function defaults to selecting the first class in the probability vector as the dominant emotion. We attribute such cases to insufficient training, which hinders the model’s ability to discern clear patterns. To address this, we extend training by increasing the number of epochs, augment the dataset, and refine label quality. These adjustments resolve the issue, ensuring that the model no longer produces ambiguous probability distributions. Figure 11 below shows the deep learning network flow diagram.

## 5. Model Training and Testing

### 5.1. Training Phase

For training, we use 80% of our dataset, divided into four sub-folders, each representing a distinct emotion category. The remaining 20% is reserved for testing. Since the emotion categories varies across the dataset, we do not evaluate the model separately on each subset to maintain consistency in testing conditions.

The driver safety monitoring model is developed in Jupyter, running on a Windows 10 system with Intel^®^ Core™ i7-4870HQ and 16.0 GB RAM. Below are the parameter settings used during the training process. Our model is trained from scratch without any pre-trained weights.

To select the parameters’ values, we first use manual research approach where we started with a baseline value for each parameter, giving the parameters default or widely recommended settings to establish a baseline. Then, we start experimenting with different values for each parameter and observing the effects on the performance, loss, accuracy, overfitting, and time consumption. Parameters’ values are chosen based on multiple attempts we conduct on each parameter. However, the need to use a more efficeint systematic approach arises. Therefore, we choose to use Bayesian optimization with validation metrics to guide tuning. This tuning is to be performed using KerasTuner, the hyperparameters’ tuning tool for automatic tuning. We install KerasTuner and import the required TensorFlow models. Then, we initialize the Bayesian Optimization Tuner and begin the search for the best model parameters. Once determined, we retrain the model with the new parameters shown in Table 5 below.

As indicated in Table 5 above, we test the system performance with different batch sizes, starting with small sizes like 16 and gradually moving to larger sizes like 64 to find the balance between stability and fast training (less time consumption). The model has a high validation accuracy and a low loss function with a batch size of 64. When using the separable Conv2d setup and lowering the batch size to 32, the validation accuracy goes up, and the loss goes down even more.

Next, for the number of epochs, which represents the number of full passes through the dataset, we tune it by observing the loss during training and testing, and then based on that, we choose the optimal number of epochs to avoid over-fitting. Then, for the learning rate, we use Adam, the adaptive optimizer for tuning. We also experience different learning rates and observe the performance metrics to choose the optimal learning rate that minimizes the loss. For the activation function, we use the commonly used ReLU (Rectified Linear Unit) activation function since it is known to be efficient in tasks that need speed and efficiency. It supports the CNN to learn relevant facial features and extract them. Also, it supports responsiveness and scalability. Therefore, it is the perfect function for our models’ needs.

On the other hand, we also use categorical cross-entropy as the loss function for our multi-class classification tasks due to its sensitivity to class differences, its ability to work efficiently in training with a softmax output layer to make the training more accurate and stable, and its simplicity. For the optimizer, we use Adam Optimizer with a learning rate of 0.001, categorical cross-entropy loss, and accuracy as the metric. The reason for choosing it is that it combines the advantages of adaptive learning rate and momentum. The learning rate of 0.001 is employed to prevent overshooting in subsequent epochs.

### 5.2. Testing Phase

To assess the model’s performance, we evaluate it on the testing set (20% of the original dataset), measuring its accuracy in predicting unseen data. This step ensures the model’s ability to generalize beyond the training examples. During evaluation, the model processes randomly selected images and predicts their classes using the predict() function, which generates a probability distribution across emotion categories. The class with the highest probability is assigned as the predicted label. Detailed results, including prediction outcomes and evaluation metrics, are presented in Section 6.

## 6. Performance Evaluation

**Figure 12:**
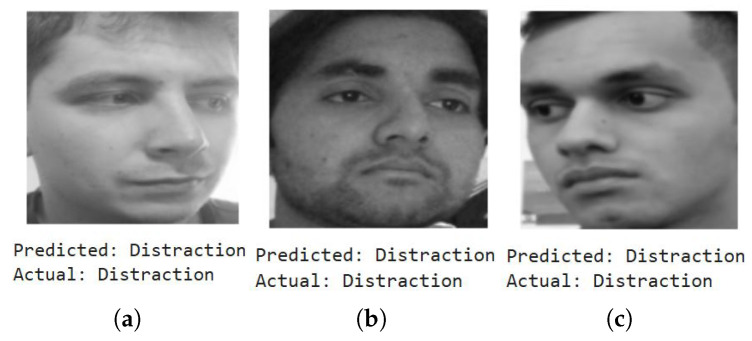
Examples of distraction detected by the model: (**a**) driver looking sideways; (**b**) driver lowering head slightly; (**c**) driver glancing away from the road. Each image shows both the predicted and actual label as "Distraction".

**Figure 13:**
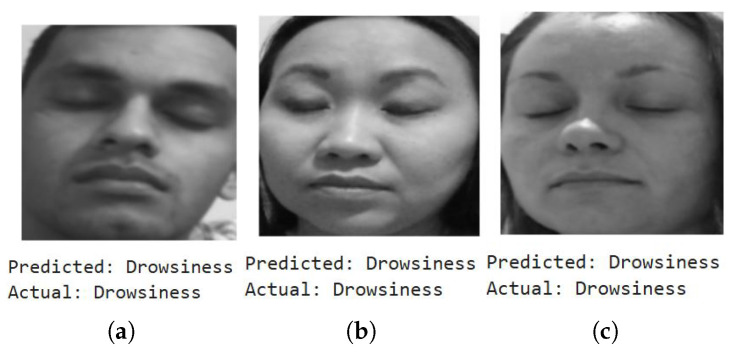
Examples of drowsiness detection by the model: (**a**) driver closing eyes briefly (early drowsiness); (**b**) driver showing partial eye closure (fatigue state); (**c**) driver with prolonged eye closure (severe drowsiness). Each image displays both the predicted and actual label as “Drowsiness”.

**Figure 14:**
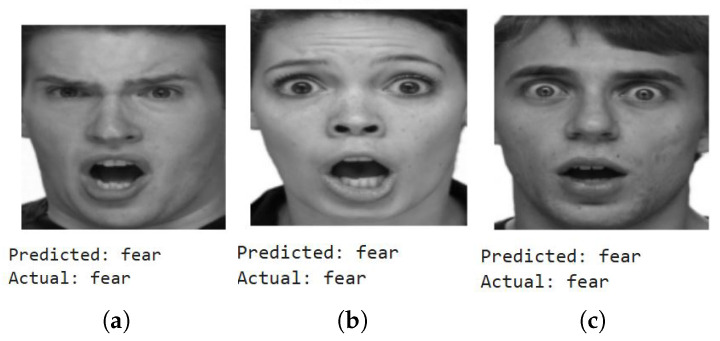
Examples of fear detection by the model: (**a**) startled facial reaction with open mouth and wide eyes; (**b**) shocked expression with raised eyebrows; (**c**) fearful reaction with tense facial features. Each image displays both the predicted and actual label as "Fear".

**Figure 15:**
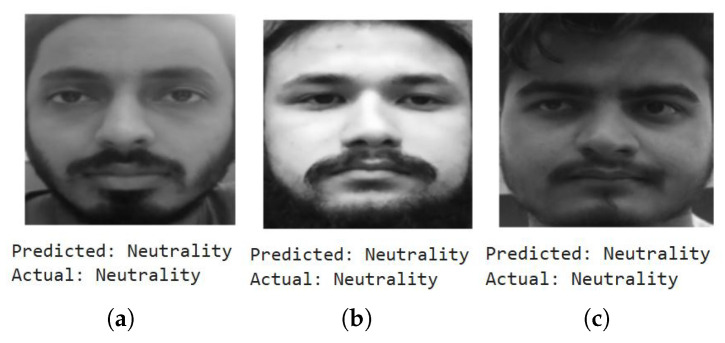
Examples of neutrality detection by the model: (**a**) with a relaxed facial expression and steady gaze; (**b**) maintaining a calm face with neutral eye contact; (**c**) presenting a composed face. Each image displays both the predicted and actual label as "Neutrality".

We first show a few cases of detecting emotions using a random subset of images generated from the testing dataset, encompassing a diverse range of emotions. When fed into the model, the results (shown below) demonstrate its superior accuracy in facial emotion classification for driver safety monitoring systems. Figure 12, Figure 13, Figure 14 and Figure 15 show the successful detection of distraction, drowsiness, fear, and neutrality by our model.

Out of 10,000 images per emotion, 20% of images (2000 images) have a Laplacian variance below the threshold of 250, indicating blurriness in around 8000 images across all emotions. This shows that our system accurately detects emotions even in challenging conditions, such as blurry/shaky or dimly lit images.

**Figure 16:**
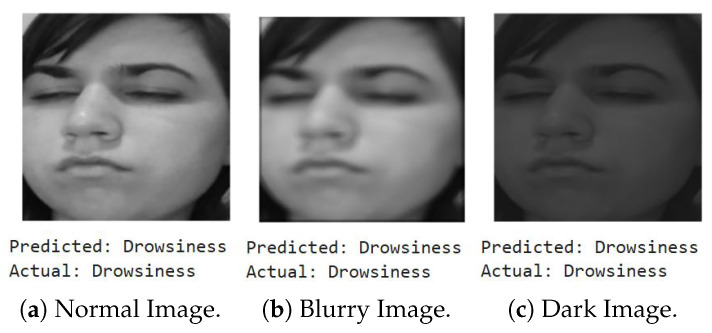
Accurate identification of drowsiness in normal, blurry, and dark images.

To validate robustness, we test a randomly selected distraction image under three conditions: original (normal settings), blurred, and dimly lit. The results confirm that our system performs reliably across varying image quality and lighting scenarios (Figure 16).

**Figure 17:**
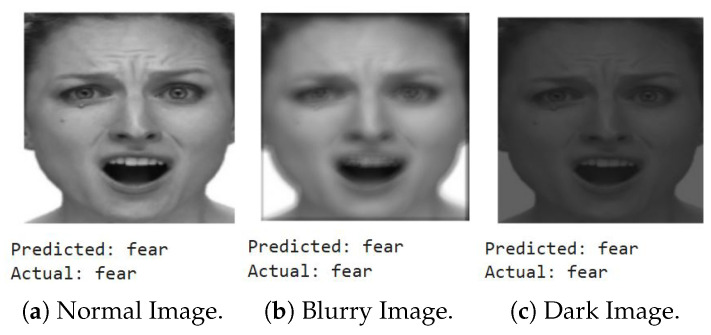
Accurate identification of fear in normal, blurry, and dark images.

We repeat the same evaluation using a randomly selected fear image, testing it under normal, blurred, and dimly lit conditions. As illustrated in Figure 17, our system consistently detects fear with high accuracy across all scenarios.

To systematically evaluate model performance, we employ multiple metrics: accuracy, loss function, confusion matrix, Receiver Operating Characteristic (ROC) curve analysis, as well as cross-entropy as a loss metric to quantify prediction errors.

**Figure 18:**
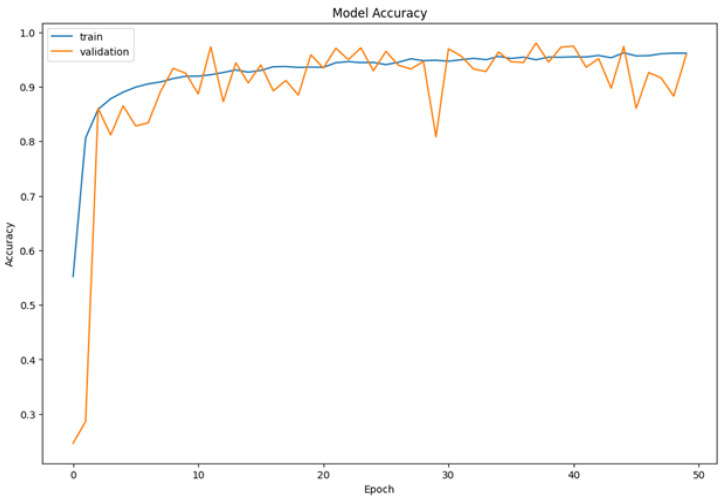
Model accuracy.

As shown in Figure 18, the model demonstrates progressive improvement in accuracy during training, ultimately achieving 98.6% overall accuracy, which notably exceeds comparable architectures in driver monitoring tasks. Our model’s performance is evaluated using overall classification accuracy, computed as the ratio of correctly predicted samples to the total number of samples in the test set as clarified in the equation below:

(6) $$\begin{array}{c} \mathrm{Accuracy}=\frac{\mathrm{Number}\_\mathrm{of}\_\mathrm{Correct}\_\mathrm{Prediction}}{\mathrm{Total}\_\mathrm{Number}\_\mathrm{of}\_\mathrm{Samples}} \end{array}$$

The training process reveals the model’s iterative weight adjustment to minimize the loss function, where additional epochs enable finer optimization and convergence toward higher predictive accuracy. Figure 18 represents the accuracy for the inception model.

**Figure 19:**
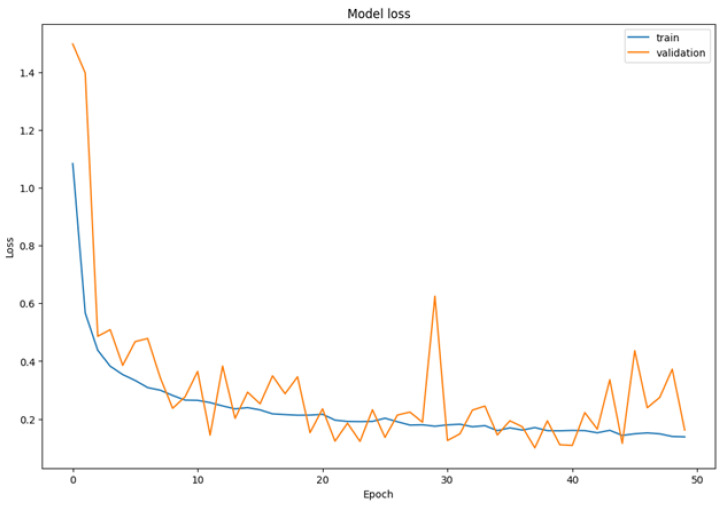
Model loss.

Figure 19 represents the loss of the inception model and demonstrates a consistent decrease in the loss function over training epochs, indicating successful optimization of the model’s classification performance. This progressive reduction in loss confirms the model’s improving ability to correctly categorize input images into their respective emotion classes.

**Figure 20:**
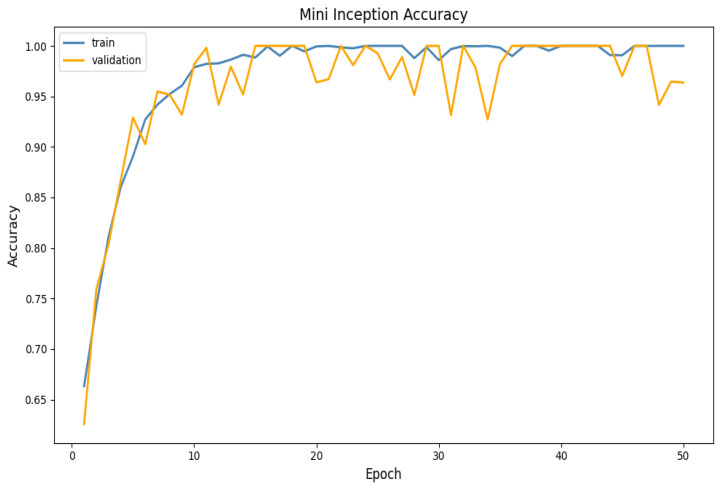
Mini-Inception accuracy.

**Figure 21:**
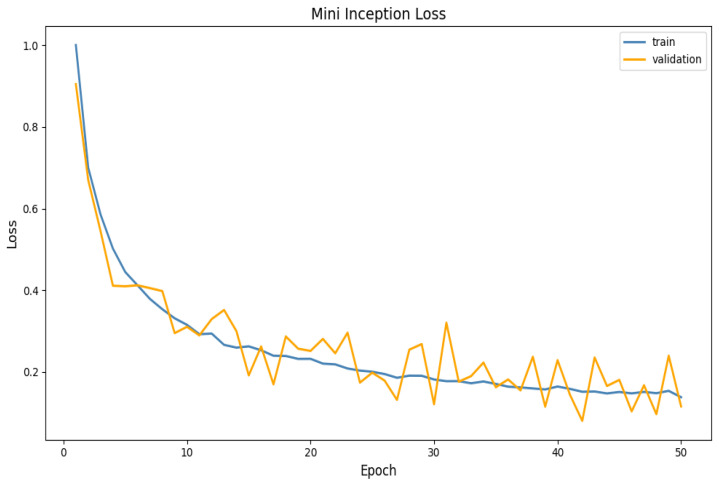
Mini-Inception loss.

The accuracy and loss functions for the Mini-Inception function are clarified in Figure 20 and Figure 21 below:

Overall, the accuracy and loss plots’ performance trends confirm that the Mini-Inception architecture’s generalization and rapid convergence are primarily driven by the multi-level feature extraction made possible by the repeated calls of the Inception model function. This, in turn, directly contributes to the model’s steady loss reduction and smooth accuracy growth.

The confusion matrix below evaluates the performance of a classification algorithm. Each row corresponds to the instances of an actual emotion class, while each column represents the predicted emotion class. The matrix includes four key metrics: (1) True Positive (TP) which is the number of correct positive predictions (i.e., the model correctly predicts the positive class), (2) False Positive (FP) which is the number of incorrect positive predictions (i.e., the model predicts positive when the actual class is negative), (3) True Negative (TN) which is the number of correct negative predictions (i.e., the model correctly identifies the negative class), and (4) False Negative (FN) which is the number of incorrect negative predictions (i.e., the model predicts negative when the actual class is positive).

**Figure 22:**
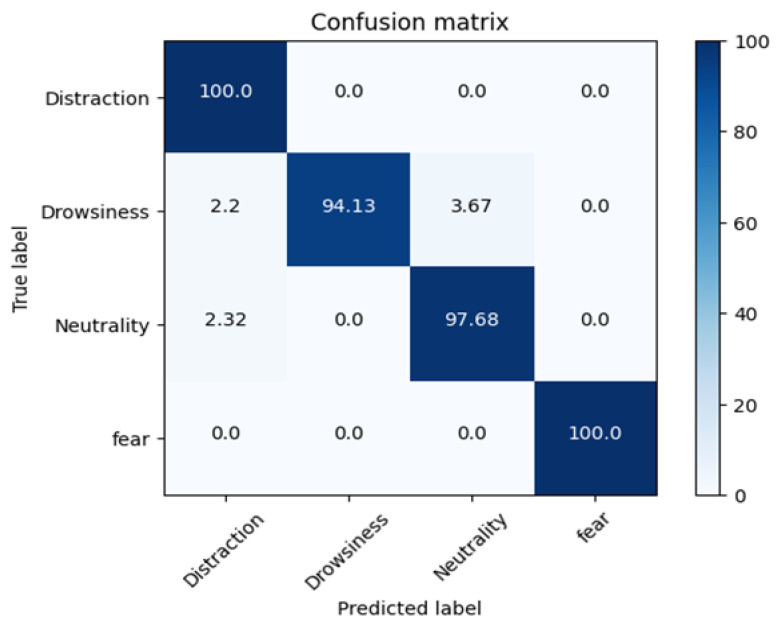
Model confusion matrix.

From Figure 22, we observe that drowsiness has the highest recognition accuracy and the least misclassification, followed by distraction, neutrality, and fear.

The confusion matrix offers a granular assessment of the model’s performance beyond basic accuracy, revealing not only the frequency of errors but also their nature (e.g., false positives vs. false negatives). These insights are essential for optimizing the classifier. Based on the confusion matrix, we calculate the precision, recall, and F1-score to further evaluate model performance.

**Figure 23:**
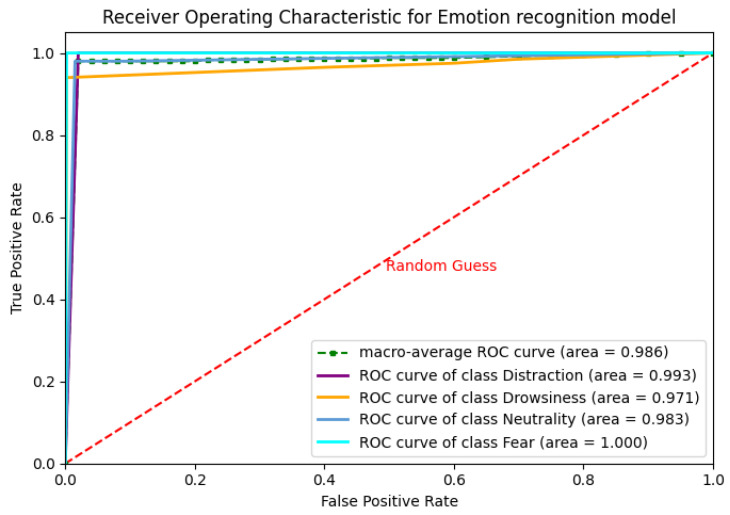
The ROC curve.

Table 6 presents the model’s performance metrics. The F1-score, which balances precision and recall via their harmonic mean, consistently exceeds 0.8 for all emotion classes, indicating robust classification performance. Precision (positive predictive value) measures the proportion of correctly predicted positive instances, while recall (sensitivity) quantifies the model’s ability to detect actual positives. Figure 23 shows the ROC curve, which evaluates classifier effectiveness by plotting the true positive rate (TPR, or recall) against the false positive rate (FPR). Here, TPR = TP/(TP + FN) represents the fraction of actual positives correctly identified, while FPR = FP/(FP + TN) indicates the proportion of negatives erroneously classified as positives.

## 7. Comparative Analysis of the Best Existing Models Against Our Model

This section compares the proposed model with four recent prominent models for driver emotion recognition [9,10,11,18]. A brief description of how these models operate is given below:

1. Jain et al. [9] depict an Automated Hyperparameter Tuned Model (AHTM) that combines Squirrel Search Optimization with Deep Learning Enabled Facial Emotion Recognition (SSO-DLFER) for face detection and emotion recognition. The model first uses RetinaNet for face detection and then applies SSO-DLFER-based Neural Architectural Search (NASNet) to extract emotions.
2. Sudha et al. [10] present the Driver Facial Expression Emotion Recognition (DFEER) system using a parallel multi-version optimizer. The main goal is to handle partial occlusions and motion variations of expressions.
3. Sukhavasi et al. [11] present a hybrid model that combines a Support Vector Machine (SVM) and CNN to detect six to seven driver emotions with different poses. The model fuses Gabor and Local Binary Patterns (LBPs) to extract features which are usedby the hybrid model for classification.
4. Hilal et al. [18] propose Transfer Learning-Driven Facial Emotion Recognition for Advanced Driver Assistance System (TLDFER-ADAS) for driver facial emotion recognition. The model uses Manta Ray Foraging Optimization (MRFO) and Quantum Dot Neural Networks (QDNNs) for emotion classification.

These models are selected for comparison based on three key criteria: (1) their focus on emotion recognition in driving contexts, (2) exclusive reliance on facial expression monitoring and analysis, and (3) methodological alignment with our objective of effective driver emotion monitoring. Like our proposed system, these models analyze emotions solely through facial features rather than other physiological or behavioral indicators. In addition, these models detect many of the emotions our model detects and use some or many common datasets we use to achieve a good comparison of accuracy. Our comparison focuses on the input dataset, preprocessing, architecture, and performance aspects.

### 7.1. Input Data

The performance of an AI-based model is highly dependent on the dataset. The amount of data, its diversity, and the ability of the model to handle the data significantly impact its performance. Table 7 compares the dataset used by the existing models with the proposed model.

The table shows that the proposed model trains on a significantly larger dataset of approximately 198,000 images and 1600 videos. In contrast, other models use relatively smaller image-based datasets. Using a large dataset reduces the chance of model overfitting. Furthermore, it improves the model’s ability to handle variable data and provides better accuracy. Another advantage of our proposed model is its training on more comprehensive datasets that capture critical driver-specific states beyond conventional emotions. While existing models primarily utilize generic facial expression datasets (containing variations of angles, basic emotions like happiness/sadness, demographics, and image quality), our model incorporates specialized behavioral features including drowsiness, microsleep episodes, distraction patterns, and naturalistic responses that are highly relevant to actual driving scenarios. This domain-specific training enables the more accurate detection of safety-critical states compared to models limited to recognizing general emotions that have less direct impact on driving performance.

### 7.2. Preprocessing Techniques

Next, we compare the preprocessing techniques used by the models. Preprocessing is critical for ensuring that the model receives the input in a consistent format and can learn relevant features without being biased or sensitive to unnecessary variations in the data. The performance of applied ML/AI models directly benefits from consistent and accurate data. Each study employs various preprocessing techniques to eliminate noise and inconsistencies from the data. Table 8 provides an overview of techniques applied by the models.

Normalization serves two primary purposes: (1) maintaining consistent pixel scaling across all input images, and (2) facilitating faster model convergence while preventing overfitting. As evidenced in the comparative table, all models incorporate normalization as a standard preprocessing step. Additionally, each model employs customized image resizing and noise suppression techniques to optimize input data quality for their respective architectures.

A critical preprocessing stage involves data unification to ensure input consistency across diverse sources. While existing models often overlook this requirement when handling multiple datasets, our approach systematically addresses format variations through standardized conversion. Using Python’s Pillow library, we transform all images to the JPEG format (.jpg), achieving three key benefits: (1) uniform compression characteristics, (2) consistent color channel representation (RGB), and (3) standardized bit depth. This format standardization maintains optimal image quality while eliminating inconsistencies from heterogeneous source formats and resolutions. Importantly, our controlled conversion process preserves a fixed compression ratio, preventing quality degradation while ensuring processing efficiency across both image and video frames.

Grayscale conversion is a critical preprocessing step when color information is non-essential. In a DMS, facial emotion analysis takes precedence over color data, making grayscale transformation beneficial for reducing image complexity and computational load. Following the approach of Sukhavasi et al. [11], we adopt this method to streamline processing. Additionally, our model incorporates contrast enhancement/normalization to mitigate variations in lighting conditions across datasets, ensuring consistent input quality. Finally, data augmentation is applied to enhance model robustness and training efficiency.

### 7.3. Architectural Differences and Performance

This section compares the architectural differences between the proposed and existing emotional detection models. Each model implements a unique combination of AI classifiers and layers to detect the driver emotions. Table 9 provides an overview of the architecture of all models.

Each study explores different combinations of classifiers, layer depths, and filter sizes to optimize emotion detection in DMS. Larger filters enable the model to classify a broader range of expressions (e.g., fatigue or distractions), while deeper layers improve recognition of subtle and complex emotions. However, this approach increases computational costs, training time, and risks overfitting on small datasets. Conversely, smaller filters with deeper layers excel at capturing fine details (e.g., brow movements) but may still demand significant resources. Shallower architectures reduce computational overhead yet struggle with complex emotion patterns. Thus, the design of DMS models involves a trade-off between accuracy which is governed by layer depth and filter size and computational efficiency.

While all evaluated models employ varying architectures (e.g., layer depths and filter sizes), each achieves an average accuracy exceeding 90%. The AHTM model leads with 99.60% accuracy, followed closely by TLDFER-ADAS (99.30%), while our proposed model ranks third at 98.6%. However, AHTM, DFEER, and TLDFER-ADAS lack real-time capability, and although the hybrid model operates in real time, its accuracy is comparatively lower. In contrast, our proposed model uniquely balances high accuracy (98.6%) with real-time performance as demonstrated in Table 10.

The proposed model offers a key advantage over existing approaches by leveraging the Inception architecture and separable convolutional layers, achieving an optimal balance between accuracy and computational efficiency. The Inception model is widely recognized for its effectiveness in deep learning tasks, particularly image classification, due to its multi-scale processing capability, which employs parallel filter sizes and max pooling operations to extract diverse features without excessive parameters. Its balanced design that combines depth (more layers) and width (more filters) enables the capture of complex patterns while maintaining computational feasibility. Further optimizations, such as batch normalization and dropout, enhance scalability and reduce overfitting, making the model suitable for deployment and large datasets. Additionally, Inception’s use of auxiliary classifiers stabilizes training and accelerates convergence. Despite newer architectures emerging, Inception remains a robust choice for demanding tasks, particularly in our model, where simplicity, computational efficiency, and high performance are critical, ensuring competitive results.

The proposed model enhances emotion recognition by employing a combination of 1 × 1, 3 × 3, and 5 × 5 convolutional layers, enabling it to classify a broader range of emotions and complex expressions more effectively than existing models, which predominantly rely on 3 × 3 convolutions. By first reducing dimensionality through a 1 × 1 convolution before applying larger kernels (3 × 3 and 5 × 5), the model significantly lowers computational costs while maintaining high accuracy. Additionally, unlike existing approaches that focus on general emotions (e.g., happiness or sadness) with limited relevance to driving, our model is specifically optimized to detect critical driving-related states such as drowsiness and distraction, making it more practical for real-world applications.

In summary, the proposed model achieves an optimal balance between complexity and computational efficiency while effectively capturing critical driving-related emotions. This combination of performance and practicality makes our approach particularly well-suited for real-world in-vehicle driver monitoring systems. Several technical issues need to be resolved for hardware based implementation of the proposed Driver Monitoring System (DMS). Specifically, this work overcomes two primary challenges to deploy an effective driver monitoring system (DMS) on the MAX78000FTHR microcontroller. First, the device’s memory limitations are addressed by employing model quantization, pruning, and a lightweight CNN architecture, which together enable real-time inference. Second, the model’s performance degradation in low-light conditions is mitigated by augmenting the training dataset with nighttime imagery. By resolving these hardware-specific constraints, we develop a dependable, low-power DMS ready for real-world automotive integration.

## 8. Hardware Implementation

We implement the proposed model on a compact, cost-effective, and energy-efficient microcontroller system designed for seamless integration into vehicles. Equipped with a real-time alert mechanism, the system detects critical emotional states—such as fear, drowsiness, or distraction—and triggers appropriate responses. Building on this foundation, our prototype is an AI-powered copilot capable of continuous emotion recognition and proactive safety interventions, thereby reducing accidents and enhancing driver support. This system holds particular promise for drivers with physical disabilities.

Our system leverages edge AI on the MAX78000FTHR microcontroller to enable low-power, real-time facial analysis—a significant advancement over conventional passive monitoring solutions. By harnessing the MAX78000’s CNN accelerator and an optimized facial recognition model, we eliminate processing delays and ensure instantaneous response. When a hazardous emotional state (e.g., drowsiness or distraction) is detected, an active buzzer immediately alerts the driver, proactively mitigating risks before accidents occur. Unlike vision-based systems that often fail under compromised driving conditions, our solution operates with milliwatt-level power consumption while delivering real-time auditory warnings. This approach overcomes key limitations of existing technologies, offering a reliable, cost-effective, and energy-efficient platform for continuous driver monitoring.

### 8.1. Microcontroller Selection

**Figure 24:**
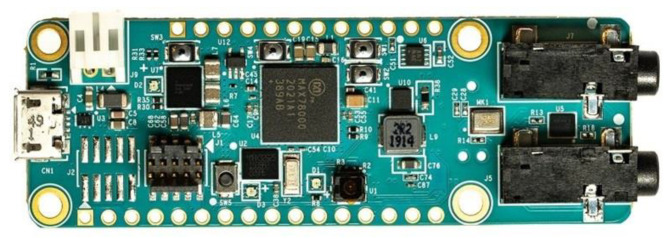
The MAX78000FTHR Microcontroller.

We select the Max78000FTHR, an AI microcontroller designed for efficient real-time data processing. This ultra-low-power device features a CNN accelerator, enabling high-performance AI computations. It utilizes dual-core processing, combining an Arm Cortex-M4 core (Arm Holdings, Cambridge, UK) with a Floating Point Unit (FPU) (Intel, Santa Clara, CA, USA) running at 100 MHz and a RISC-V coprocessor (SiFive, Santa Clara, CA, USA) operating at 60 MHz, ensuring fast and efficient computation for our application. Figure 24 presents the microcontroller.

The MAX78000FTHR is a cost-effective, portable, and ultra-low-power microcontroller with broad compatibility across peripherals and systems. Its capabilities make it ideal for diverse applications, including (1) facial recognition; (2) audio processing (multi-keyword recognition, sound classification, noise cancellation); (3) time-series data analysis (heart rate monitoring, multi-sensor fusion, predictive maintenance); and (4) object detection and classification.

Given its versatility and efficiency, the Max78000FTHR is the optimal choice for our model. Table 11 summarizes its key features, while further details can be found in [57].

#### CNN Accelerator

The MAX78000FTHR microcontroller integrates a hardware-based CNN accelerator, enabling energy-efficient inference for deep learning models. This dedicated neural network engine processes CNNs with exceptional power efficiency, consuming up to 100x less power than general-purpose microcontrollers—as low as 1 mW. By offloading AI computations from the Central Processing Unit (CPU) and enabling parallel processing of CNN layers, it drastically reduces inference latency while maintaining ultra-low power consumption. This ensures seamless execution of always-on AI applications in the background.

The accelerator optimizes memory usage with 442 KB of weight memory and 512 KB of data memory, eliminating dependency on external RAM for model execution. Additionally, it supports TensorFlow and PyTorch, allowing seamless deployment of pre-trained models via Maxim’s AI Model Converter. This tool also facilitates model quantization, further enhancing efficiency without compromising performance.

Having this CNN accelerator affects the performance of our model tremendously. The parallel computation for the CNN layers leads to analyzing drivers’ facial emotions from the camera frames in real time without delays, which is crucial for driver safety. Fast real-time inference is significant to ensure fast face detection, real-time monitoring, and fast responses and emergency actions to detected alarming emotions that indicate possible threats to drivers’ lives. The ultra-low power consumption is crucial for our in-vehicle model, where energy efficiency matters. It allows continuous facial monitoring without power drainage or heating up. Another important benefit of this accelerator is the Edge AI processing that requires no internet, which eliminates the delay that could be caused by cloud processing and saves us more response time, leading to detecting driver faces instantly without network latency and to working in remote areas or in tunnels where network connection can be lost. On the other hand, having this accelerator with optimized memory usage that only fits small models leads us to use Maxim’s model converter to quantize our model and compress it while maintaining detection accuracy.

### 8.2. Components and Sensors Integration

■ **A built-in CMOS VGA Image Sensor (Camera):** This microcontroller allows image processing on the board and the direct processing of grayscale images to save memory and power. It has the Complementary Metal Oxide Semiconductor (CMOS) Video Graphics Array (VGA) image sensor that works as a camera with low power consumption. It is also called OV7675. The maximum frame rate the camera can achieve under optimal conditions is 30 frames per second (FPS). Its VGA resolution is 640 × 480 pixels, and it has a Parallel Camera Interface (PCIF) of a 12-bit interface that facilitates transferring data quickly to the microcontroller when performing real-time image processing [58].
■ **A built-in microphone:** This microcontroller has a digital microphone (SPH0645LM4H-B), which reduces the surrounding noise and enhances signal quality.
■ **A built-in Wi-Fi:** This microcontroller has a Secure Digital Input Output (SDIO) interface, which facilitates the connection of Wi-Fi modules such as the ESP8266 or ESP32. We can connect a Feather-compatible Wi-Fi board to the Feather headers. This allows sending and receiving data to and from the Alexa-based alarm system.
■ **A built-in API (Application Programming Interface):** The SDK (Software Development Kit 1.0.1) in this microcontroller provides APIs for deep learning inference, GPIO, I2C, SPI, UART, camera interfaces, and more through a set of libraries.
■ **A FeatherWing Touchscreen:** This Thin Film Transistor (TFT) screen is 2.4 inches with 240 × 320 pixels to show detected faces and emotions.
■ **An Active Buzzer:** This DC 3 V active buzzer is connected to the microcontroller and is used to produce the alarm sound when an alarming emotion is detected.

The MAX78000FTHR microcontroller executes our facial emotion recognition model, optimized for embedded deployment. Upon detecting critical emotional states (e.g., drowsiness or distress), the system triggers real-time auditory alerts via an active buzzer, providing immediate warnings to drivers. This closed-loop response ensures timely intervention for safety-critical scenarios.

### 8.3. Microcontroller Configuration

**Figure 25:**
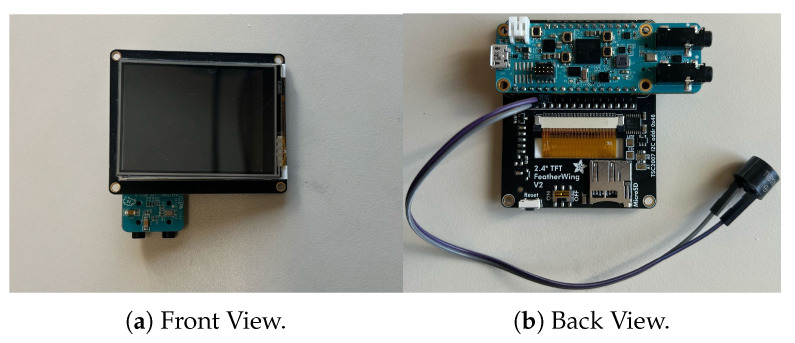
Microcontroller setup.

The proposed model was developed in Python 3.13.0 for training, then converted to C and optimized through quantization and AI8x-Synthesis, a specialized toolchain for deploying neural networks on MAX78000 microcontrollers. Quantization reduces model precision to enhance efficiency, while AI8x-Synthesis facilitates hardware-aware implementation. The final model was flashed and debugged on the microcontroller using the Visual Studio debugger within Windows Subsystem for Linux (WSL). The experimental setup is illustrated in Figure 25.

### 8.4. System Flow

**Figure 26:**
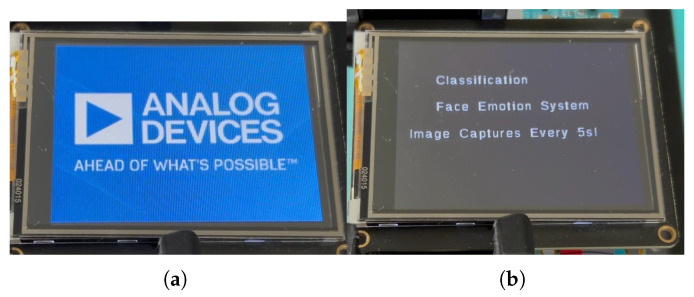
The screen output. (**a**) Startup display showing the Analog Devices logo during device initialization. (**b**) Operational screen showing the classification mode of the Face Emotion System.

Upon powering the microcontroller via the USB connection, the touchscreen initializes and displays a “Face Emotion System” splash screen as illustrated in Figure 26.

**Figure 27:**
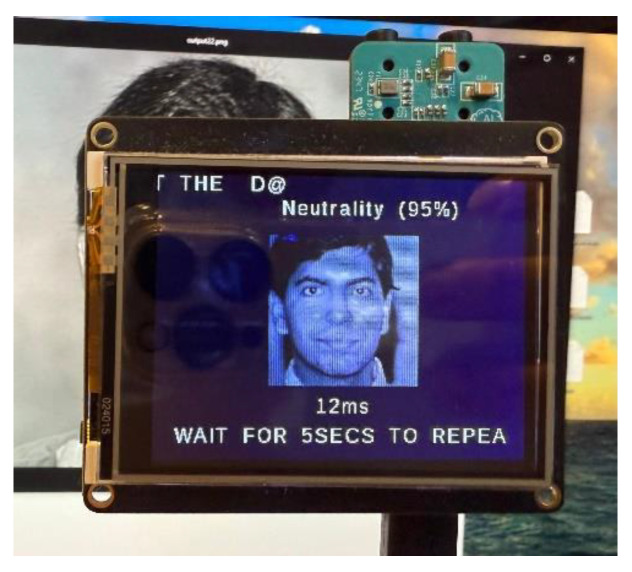
An emotion detection example.

Then, the camera activates and begins the real-time monitoring of the driver’s face. The live feed is displayed on the touchscreen, with the system capturing facial images at 5-s intervals. Each captured frame is processed by the microcontroller to predict the dominant emotion, which is then displayed on-screen along with its confidence percentage (see example in Figure 27).

When drowsiness, fear, or distraction is detected, the system triggers an auditory alert via the active buzzer to immediately warn the driver. For neutral expressions, monitoring continues uninterrupted until another safety-relevant emotion is identified.

### 8.5. Performance Demonstration

The system demonstrates robust performance on the microcontroller, achieving high-accuracy face detection and emotion recognition while maintaining real-time operation. Table 12 below presents the quantitative metrics for our model’s performance.

**Figure 28:**
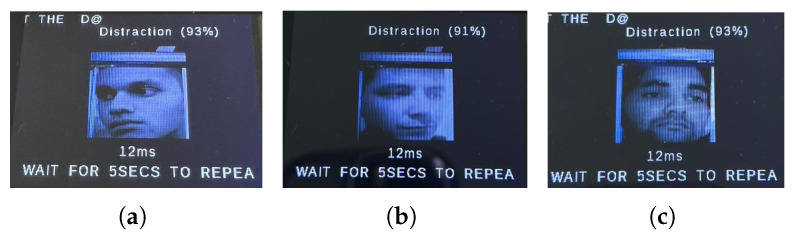
A test on random distraction images. (**a**) Distraction successfully detected with 93% confidence; (**b**) Distraction successfully detected with 91% confidence; (**c**) Distraction successfully detected with 93% confidence.

**Figure 29:**
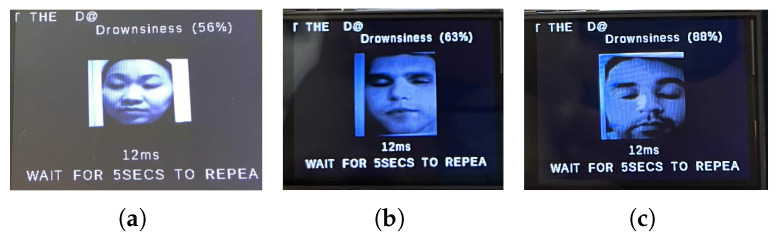
A test on random drowsiness images. (**a**) Drowsiness successfully detected with 56% confidence; (**b**) Drowsiness successfully detected with 63% confidence; (**c**) Drowsiness successfully detected with 88% confidence.

**Figure 30:**
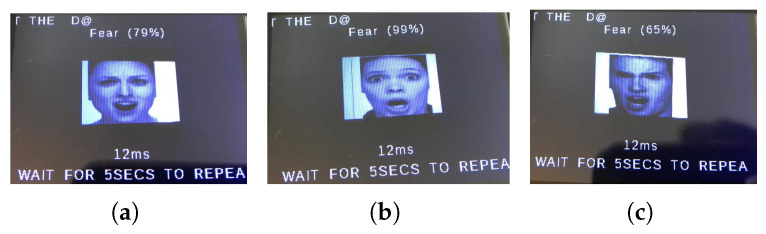
A test on random fear images. (**a**) Fear successfully detected with 79% confidence; (**b**) Fear successfully detected with 99% confidence; (**c**) Fear successfully detected with 65% confidence.

**Figure 31:**
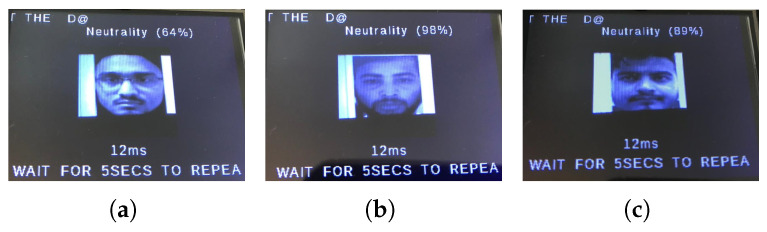
A test on random neutral images. (**a**) Neutrality successfully detected with 64% confidence; (**b**) Neutrality successfully detected with 98% confidence; (**c**) Neutrality successfully detected with 89% confidence.

During testing, the active buzzer reliably emits clearly audible alerts (approximately 85 dB) when detecting safety-critical states such as fear, distraction, or drowsiness. As evidenced in Figure 28, Figure 29, Figure 30 and Figure 31, the system successfully classifies various emotional states across diverse real-world scenarios, validating its practical effectiveness for driver monitoring applications.

**Figure 32:**
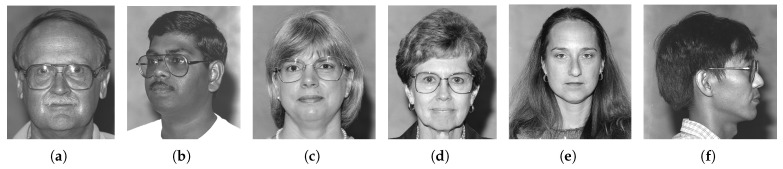
Individuals of different demographics. (**a**) Older male with glasses; (**b**) Adult male with mustache and glasses; (**c**) Middle-aged female with glasses and short hair; (**d**) Older female with glasses; (**e**) Young female with long hair and without glasses; (**f**) Young male without glasses.

**Figure 33:**
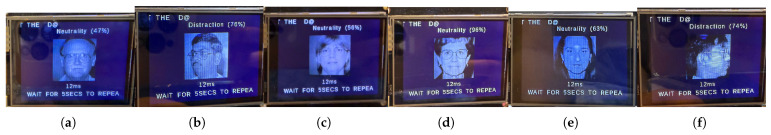
Accurate detection of emotions in individuals of different demographics. (**a**) Neutrality successfully detected; (**b**) Distraction successfully detected; (**c**) Neutrality successfully detected; (**d**) Neutrality successfully detected; (**e**) Neutrality successfully detected; (**f**) Distraction successfully detected.

**Figure 34:**
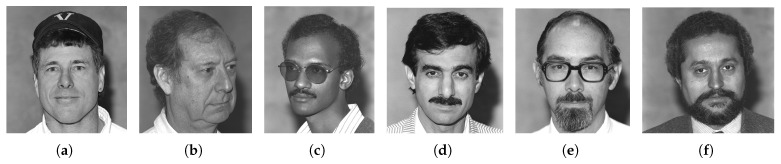
Individuals of different demographics. (**a**) Adult male wearing a cap, clean-shaven; (**b**) Older male without glasses or facial hair; (**c**) Adult male with sunglasses and mustache; (**d**) Adult male with mustache, no glasses; (**e**) Adult male with glasses, mustache, and beard; (**f**) Adult male with beard and mustache, no glasses.

**Figure 35:**
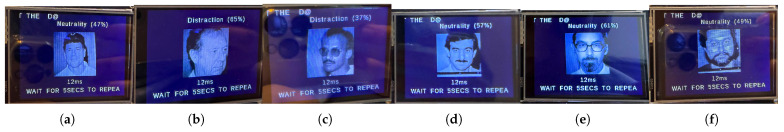
Accurate detection of emotions in individuals of different demographics. (**a**) Neutrality successfully detected; (**b**) Distraction successfully detected; (**c**) Distraction successfully detected; (**d**) Neutrality successfully detected; (**e**) Neutrality successfully detected; (**f**) Neutrality successfully detected.

As mentioned earlier, the dataset encompasses significant variation in demographics, age, and physical features, including accessories like hats and sunglasses, hair and beard styles, and dress styles. Our system maintains high emotion detection accuracy across all these groups, confirming its demographic robustness. Figure 32, Figure 33, Figure 34 and Figure 35 demonstrate our model’s ability to accurately recognize emotions across individuals of different demographics.

## 9. Comparison with Existing Driver Alert Systems

This section presents a comparative analysis between our model and existing voice-based driver alert systems, which primarily rely on facial expression analysis for safety prediction. We compare our system with existing driver alert systems across these key metrics: hardware used, processing efficiency, detection and alert methods, and latency. Table 13 summarizes the key differences across critical performance metrics.

Anil K. Biswal et al. [59] developed an IoT-based drowsy driver detection system using Eye Aspect Ratio (EAR) analysis. While their approach demonstrates effective blink detection, it exhibits three key limitations: (1) reliance on single-modal eye-region analysis while ignoring other facial features, (2) dependency on Pi cameras that perform poorly in low-light conditions, and (3) requirement for Internet connectivity to send email alerts. In contrast, our system addresses these shortcomings through multi-region facial analysis, robust low-light performance, and Short Message Service (SMS)-based alerts that maintain functionality. While most driver monitoring systems rely on in-cabin alerts, our addition of SMS-based external notifications extends safety beyond the vehicle. SMS provides a globally compatible, low-cost solution that operates independently of internet connectivity, making it ideal for remote areas. Unlike app-based notifications that require specific software, SMS works on any mobile device, ensuring wide availability and immediate alerting of emergency responders during critical events.

The Multistage End-to-End Driver Drowsiness Alerting System proposed by Sowmya-shree et al. [60] employs an escalating alert protocol involving voice warnings, seat vibrations, and robotic arm interventions. While comprehensive, this approach presents several practical limitations: (1) the multi-stage alert system may become intrusive and potentially distract drivers, (2) fingerprint verification is prone to false alarms that could trigger unnecessary escalations, and (3) the system’s complexity results in higher costs and potential latency issues that may compromise accident prevention. In contrast, our solution prioritizes simplicity and affordability while maintaining effectiveness. The streamlined design of our alert system minimizes response delays and eliminates unnecessary driver interactions, thereby providing more reliable protection without compromising safety.

Finally, we examine the emotionally adaptive driver voice alert system for Advanced Driver Assistance Systems (ADASs) developed by Sharath Yadav et al. [61]. While their system demonstrates adaptive voice alerts in response to detected emotions, it has two critical limitations: (1) the alerts remain static rather than employing dynamic conversational interaction, and (2) the system lacks real-world emergency response capabilities. Unlike their approach, our solution provides comprehensive emergency support through automated 911 calling, Global Positioning System (GPS) location sharing via SMS, and emergency contact notification—features that bridge the gap between emotion detection and tangible assistance during critical situations.

We have compared our system with existing voice-based emergency alert systems across five key metrics: accuracy, cost, portability, power consumption, and emotion detection range (Table 14). Our proposed model leverages the MAX78000FTHR’s edge AI capabilities to achieve low-power, real-time inference while detecting critical emotional states that may indicate life-threatening situations. In contrast, Raspberry Pi-based systems exhibit slower processing speeds, while robotic arm implementations suffer from mechanical complexity and latency. Furthermore, while existing systems typically focus solely on drowsiness detection, and the ADAS solution detects multiple emotions at higher hardware complexity and cost, our approach maintains an optimal balance between detection capability and system efficiency.

## 10. Conclusions

This work presents a Driver Monitoring System for real-time emotion detection, utilizing a CNN-based Inception model architecture to simultaneously enhance detection accuracy and optimize processing efficiency. The system was trained on an extensive multimodal dataset comprising approximately 198,000 images and 1600 videos from nine public and private sources, offering superior diversity that better represents real-world driving conditions compared to existing solutions.

The implementation incorporates several key preprocessing optimizations: (1) pixel normalization and noise reduction for enhanced data quality, (2) image standardization using Python’s Pillow library to handle heterogeneous dataset formats, and (3) data augmentation with grayscale conversion to both expand training samples and reduce computational complexity.

We evaluated the model’s performance using four key metrics: accuracy, F1-score, precision, and recall. The system achieved exceptional performance, with 98.6% classification accuracy which is comparable to state-of-the-art DMS implementations while maintaining significantly lower computational requirements. Across all four detected emotional states, the model demonstrated consistently strong performance, with mean values of 0.980 precision, 0.979 recall, and a corresponding F1-score of 0.979, indicating robust balance between false positives and false negatives in real-world detection scenarios.

The optimized model was subsequently deployed on the MAX78000FTHR microcontroller using the built-in camera, achieving real-time emotion detection capabilities. When hazardous states are identified, the system triggers an immediate auditory alert via buzzer activation, providing timely warnings to potentially compromised drivers.

Compared to existing techniques, our DMS demonstrates particular effectiveness in detecting critical driver states including drowsiness, distraction, fear, and natural facial expressions, all of which significantly impact driving behavior.

The proposed model offers three key advantages over existing approaches: (1) architectural innovation through the integration of an Inception model with separable convolutional layers, achieving an optimal balance between accuracy (98.6%) and computational efficiency; (2) implementation of an initial 1 × 1 convolutional layer that reduces dimensionality before subsequent 3 × 3 and 5 × 5 convolutions; and (3) specific emotion detection focused on driving-critical states (drowsiness and distraction) rather than general affective states. This targeted approach provides more actionable insights for driver monitoring systems. Finally, the system’s training on diverse datasets ensures accurate emotion detection even with shaky input (e.g., bumpy roads), while maintaining consistent performance across ages and cultures, making it ideal for real-world vehicle integration.

## Figures and Tables

**Table 1:** Previous studies on emotion recognition through facial expressions.

Ref.	Classifier Methods	Face Detectors	Datasets Names	Samples Counts	Emotions Classes	Accuracy	Blur Images Support	Dark Images Support
[9]	SSO-DLFER(GRU) ^1^	Retina-Net	KDEF ^2^	5000	Happy, sad, surprised, angry, disgusted, afraid, and neutral	99.69%	NA ^19^	NA
			KMU-FED ^3^	55 videos	Various changes in illumination (front, left, right, and back light) and partial occlusions	99.50%		
[10]	VGGNet ^4^	-	KMU-FED	55 videos	Various changes in illumination (front, left, right, and back light) and partial occlusions	95.92%	Yes	NA
			CK+ ^5^	981	Anger, Disgust, Fear, Happiness, Sadness, Surprise, Neutral, Contempt	94.78%		
[11]	CNN+SVM ^6^	Haar Features Cascade	FER2013 ^7^	32,298	Angry, Disgust, Fear, Happy, Sad, Surprise, Neutral	84.41%	Yes	NA
			CK+	981	Anger, Disgust, Fear, Happiness, Sadness, Surprise, Neutral, Contempt	95.05%		
			KDEF ^8^	5000	Happy, sad, surprised, angry, disgusted, afraid, and neutral	98.57%		
			KMU-FED	55 videos	Various changes in illumination (front, left, right, and back light) and partial occlusions	98.64%		
[12]	CogEmo-Net (CNN) ^9^	MT-CNN ^10^	CK+	981	Anger, Disgust, Fear, Happiness, Sadness, Surprise, Neutral, Contempt	0.907%	NA	NA
			DEFE+ ^11^	NA ^12^	Anger and happiness	0.351		
[13]	Squeeze-Net 1.1	-	CK+	981	Anger, Disgust, Fear, Happiness, Sadness, Surprise, Neutral, Contempt	86.86%	Yes	NA
			KDEF	5000	Happy, sad, surprised, angry, disgusted, afraid, and neutral	91.39%		
			FER2013	32,298	Angry, Disgust, Fear, Happy, Sad, Surprise, Neutral	61.09%		
			KMU-FED	55 videos	Various changes in illumination (front, left, right, and back light) and partial occlusions	95.83%		
[14]	Custom CNN + VGG16	-	FER2013	32,298	Angry, Disgust, Fear, Happy, Sad, Surprise, Neutral	68.34%	Yes	NA
[15]	Lightweight RMXception	DLib ^13^	FER2013	32,298	Angry, Disgust, Fear, Happy, Sad, Surprise, Neutral	73.32%	Yes	NA
[16]	CNN	DLib	FER2013	32,298	Angry, Disgust, Fear, Happy, Sad, Surprise, Neutral	83%	Yes	NA
[17]	Lightweight SICNET+RCNET ^14^	-	FER2013	32,298	Angry, Disgust, Fear, Happy, Sad, Surprise, Neutral	69.65%	Yes	NA
[18]	TLDFER-ADAS (QDNN) ^15^	-	FER2013	32,298	Angry, Disgust, Fear, Happy, Sad, Surprise, Neutral	99.31%	Yes	NA
			CK+	981	Anger, Disgust, Fear, Happiness, Sadness, Surprise, Neutral, Contempt	99.29%		
[19]	FERDER-net -Xception	Inspired deep cascaded multitask framework	On-road driverFE dataset	12 videos	Anger, Happiness, Sadness, Disgust, Contempt, Surprise	0.966	NA	NA
[20]	ViT ^16^	Viola and Jones	CK+	981	Anger, Disgust, Fear, Happiness, Sadness, Surprise, Neutral, Contempt	0.6665 recognition rate	NA	NA
			KMU-FED	55 sequences of images	Various changes in illumination (front, left, right, and back light) and partial occlusions	-		
[21]	Improved YOLOv8 ^17^ and Multi-Head Self-Attention (MHSA)	YOLOv8 and CNN	Distracted Driving Behavior Dataset	20	Drinking, Smoking, Phone usage	mAP ^18^ = 0.814	NA	NA
			FER2013	32,298	Angry, Disgust, Fear, Happy, Sad, Surprise, Neutral	mAP = 0.733		
This work (2024)	CNNs	DNN ^19^	Face Expression Recognition Dataset	35,900	Angry, disgust, fear, happy, neutral, sad, surprise	98.6%	Yes	Yes
			The Driver Drowsiness Dataset (DDD)	41,790	Drowsy and non-drowsy			
			Driver-drowsy Dataset	66,000	Drowsy and not Drowsy			
			Frame Level Driver Drowsiness Detection (FL3D)	53,331	Alert, microsleep, and yawning			
			Traindata Dataset	1218	Distraction, multi-face, no face, normal, object			
			Japanese Female Facial Expressions (JAFFE)	213 Images	Neutral, Happy, Angry, Disgust, Fear, Sad, Surprise			
			The Ryerson Audio-Visual Database of Emotional Speech & Song (RAV-DESS)	1440 Videos	Calm, Happy, Sad, Angry, Fearful, Surprise, and Disgust			
			Synthetic Distracted Driving (SynDD1)	6 videos (each is about 10 min long) for each participant	Distracted activities and gaze zones			
			UTA-RLDD ^20^ Dataset	30 h of RGB videos	Alert, low-vigilant, drowsy			
			FERET Dataset ^21^	PPM Images	Distracted, happy, neutral			

^1^ SSO- DLFER (GRU): Squirrel Search Optimization with Deep Learning Enabled Facial Emotion Recognition. ^2^ KDEF: Kenya Drylands Education Fund. ^3^ KMU-FED: Keimyung University Facial Expression of Drivers. ^4^ VGGNet: Visual Geometry Group. ^5^ CK+: The Extended Cohn-Kanade. ^6^ SVM: A Support Vector Machine. ^7^ FER2013: Facial Expression Recognition 2013 Dataset. ^8^ KDEF: The Karolinska Directed Emotional Faces. ^9^ CNN: A Convolutional Neural Network. ^10^ MTCNN: Multi-task Cascaded Convolutional Network. ^11^ DEFE+: Driver Emotion Facial Expression (DEFE) Dataset. ^12^ NA: Not Available. ^13^ DLib: A General Purpose Cross-platform Software Library. ^14^ Lightweight SICNET + RC-NET: Selective Inter-slice Context Network + A region-level context network for hyperreflective dots segmentation in retinal OCT images. ^15^ TLDFER-ADAS (QDNN): Transfer Learning Driven Facial Emotion Recognition for Advanced Driver Assistance System. ^16^ ViT: Vision Transformers. ^17^ YOLO8: Deep Neural Network. ^18^ mAP: Mean Average Precision. ^19^ DNN: Deep Neural Network. ^20^ UTA-RLDD: University of Texas at Arlington Real-Life Drowsiness Dataset. ^21^ FERET: Facial Recognition Technology.

**Table 2:** Comparison of our proposed DMS with existing DMS products.

Product	High Precision	Blurry Image Stabilization	Night Vision Capability	Robustness to Lighting Changes	Power Effectiveness	Indirect Face Detection	Facial Features	Unobtrusive
Seeing-Machines’ FaceAPI [23]	Yes	Yes	Yes	No	Yes	Yes	Yes	Yes
Jungo’s CoDriver [24]	Yes	No	No	No	No	No	Yes	Yes
Xperi’s FotoNation [25]	No	No	No	No	No	No	No	No
Edge3 Technologies [26]	Yes	No	No	Yes	Yes	No	Yes	Yes
Denso Driver Attention Monitor [27]	Yes	No	No	No	No	Yes	Yes	Yes
Ellice Healthy Eyewear [28]	Yes	No	No	No	Yes	No	Yes	No
Optalert [29]	Yes	No	No	Yes	No	No	No	Yes
Gentex Full Display Mirror [30]	No	No	No	No	No	No	Yes	Yes
Smart Eye Pro [31]	Yes	No	Yes	Yes	Yes	No	Yes	Yes
ZF CoDriver [32]	Yes	No	No	Yes	Yes	No	Yes	Yes
Omni-Vision OV2311 [33]	No	Yes	No	No	No	No	No	Yes
Denso Driver Status Monitor [34]	No	No	No	No	Yes	Yes	Yes	No
Autoliv Driver Facial Monitoring Camera [35]	No	No	No	No	Yes	No	No	No

**Table 3:** Driver’s face characteristics for each emotion.

Emotion	Eye Features	Mouth Features	Head Cues	Temporal Pattern
Fear	wide-open eyes	wide-open mouth	forward-facing head	Sudden changes
Distraction	open eyes	closed mouth	tilted or rotated head	Intermittent shifts
Drowsiness	closed or semi-closed eyes	closed or open mouth	Nodding, slow head tilt	Gradual slow progression
Neutral	Normal openness	Relaxed or closed	Balanced, steady, and forward-facing head	Stable baseline

**Table 4:** Data sources used in creating datasets for our work.

Dataset	Accessibility	Data Format	Emotion	Participant Count	Sample Count	Data Size	Estimated Download Frequency
Face Expression Recognition Dataset [41]	Public	JPEG	Angry, disgust, fear, happy, neutral, sad, surprise	NA	35.9 k	126 MB	More than 53.9 k
Driver Drowsiness Dataset (DDD) [42]	Public	PNG	Drowsy and non-drowsy	NA	41.8 k	3 GB	More than 5767
Driver Drowsiness Dataset [43]	Public	PNG	Drowsy and not drowsy	NA	66.5 k	3 GB	More than 70
Frame Level Driver Drowsiness Detection (FL3D) [44]	Public	JPEG	Drowsiness (alert, microsleep, and yawning)	NA	53.3 k	628 MB	More than 445
Traindata Dataset [45]	Public	JPEG	Distraction, multi-face, No face, normal, and object	NA	1218	239 MB	More than 8
Japanese Female Facial Expressions (JAFFE) [46,47,48]	Public	TIFF	Neutral, happy, angry, disgust, fear, sad, surprise	10	213	12.2 MB	More than 11 k
The Ryerson Audio-Visual Database of Emotional Speech and Song (RAVDE-SS) [49,50]	Public	WAV Videos	Calm, happy, sad, angry, fearful, surprise, and disgust	24	1440 Videos	24.8 GB	More than 50.2 k
Synthetic Distracted Driving (SynDD1) Dataset [51,52]	Private	MP4 Videos	Distracted Activities, and Gaze Zones	10	60 Long Videos	48.1 GB	NA
UTA-RLDD Dataset [53,54]	Private	MP4, MOV, M4V Videos	Drowsiness (alert, sleepy, very sleepy)	30	90 Total Videos (3 Videos for Each)	120 GB	NA
FERET Dataset [55,56]	Private	PPM Images	Distracted, happy, neutral	NA	around 5000 Total Images	8.5 GB	NA

**Table 5:** Training parameters settings.

The Parameters	Their Values
Image size	240 × 240 pixels
Optimizer	Adam
Loss Function	Categorical Cross-entropy
Activation Function	ReLU
Batch Size	32
Learning Rate	0.001
Measuring Metric	Accuracy

**Table 6:** F1 score, precision, and recall.

	Distraction	Drowsiness	Neutrality	Fear
F1_score	0.978579	0.969773	0.971165	1.0
Precision	0.958057	1.000000	0.965596	1.0
Recall	1.000000	0.941320	0.976798	1.0

**Table 7:** Input dataset comparison between the proposed model and existing models.

Model	Dataset(s)	Samples
Hybrid [11]	FER2013 ^1^, CK+ ^2^, KDEF ^3^, KMU-FED ^4^	41,500 images (Approx)
AHTM ^5^ [9]	KDEF, KMU-FED	6000 images (Approx)
DFEER ^6^ [10]	CK+, KMU-FED	1500 images (Approx)
TLDFER-ADAS ^7^ [18]	FER2013, CK+	36,000 images (Approx)
Proposed Model	FER2013, DDD ^8^, Driver-drowsy dataset, FL3D ^9^, Traindata Dataset, JAFFE ^10^, RAVDESS ^11^, SynDD1 ^12^, UTA-RLDD ^13^ Dataset, FERET Dataset ^14^	Overall: 198,000 images (Approx) + 1600 videos (Approx)

^1^ FER2013: Facial Expression Recognition 2013 Dataset. ^2^ CK+: Extended Cohn-Kanade. ^3^ KDEF: Karolinska Directed Emotional Face. ^4^ KMU-FED: Keimyung University Facial Expression of Drivers. ^5^ AHTM: Adaptive Hierarchical Transformer with Memory model. ^6^ DFEER: Data Extraction and Enrichment Framework. ^7^ TLDFER-ADAS: Advanced Driver Assistance Systems. ^8^ DDD: Driver Drowsiness Dataset. ^9^ FL3D: Frame Level Driver Drowsiness Detection. ^10^ JAFFE: Japanese Female Facial Expressions. ^11^ RAVDESS: The Ryerson Audio-Visual Database of Emotional Speech and Song. ^12^ SynDD1: Synthetic Distracted Driving. ^13^ UTA-RLDD: The University of Texas at Arlington Real-Life Drowsiness Dataset. ^14^ FERET: The Face Recognition Technology program.

**Table 8:** A comparison of preprocessing techniques between proposed model and existing models.

Model	Normalization	Format Unification	Grayscale Conversion	Contrast Enhancement	Data Augmentation
Hybrid [11]	Yes	No	Yes	Yes	No
AHTM [9]	No	No	No	No	No
DFEER [10]	Yes	No	No	No	No
TLDFER-ADAS [18]	Yes	No	No	Yes	No
Proposed Model	Yes	Yes	Yes	Yes	Yes

**Table 9:** Architectural differences between proposed and existing models.

Model	Classifier	Layer Type	Filter Size/Stride	Filters Quantity	Output Size	Accuracy
Hybrid [11]	CNN+SVM ^1^	Conv1	3 × 3/1	8	-	94.20%
		Maxpool1	2 × 2/1	8	-	
		Conv2	3 × 3/1	16	-	
		Maxpool2	2 × 2/1	16	-	
		Conv3	3 × 3/1	32	-	
		FC1 ^2^	-	-	1000	
		FC1	-	-	7	
AHTM [9]	SSO-DLFER (GRU) ^3^	Average-Pooling	-	-	-	99.60%
		Identity Mapping	-	-	-	
		Conv	-	-	-	
		Separable Conv	-	-	-	
		MaxPooling	-	-	-	
		Encoder	-	414	-	
		Decoder	-	-	-	
		WELM ^4^ Classifier	-	-	-	
DFEER [10]	VGGNet ^5^	Conv1	3 × 3/1	2	64	95.35%
		Maxpool1	3 × 3/1	1	64	
		Conv2	3 × 3/1	2	128	
		Maxpool2	3 × 3/1	1	128	
		Conv3	3 × 3/1	3	256	
		Maxpool3	3 × 3/1	1	256	
		Conv4	3 × 3/1	3	512	
		Maxpool4	3 × 3/1	1	512	
		Feature Fusion	3 × 3/1	1	1024	
		Conv1	3 × 3/1	1	512	
		Feature Fusion	1 × 1/1	1	1	
		Conv2	-	-	-	
		Feature Fusion	-	-	-	
		Conv3	-	-	-	
TLDFER ADAS [18]	TLDFER-ADAS (QDNN ^6^)	Xception Conv1	3 × 3/1	-	-	99.30%
		Xception Conv2	3 × 3/1	-	-	
		Depth-wise Conv Layer	3 × 3/1	-	-	
		Pointwise Conv Layer	1 × 1/1	-	-	
Proposed Model	Inception-Model (CNNs)	Convolutional Layer	1 × 1/1	64	Vector of length four ^7^	98.6%
		Convolution Kernel	1 × 1/1	96		
		Separable Convolutional Layer	3 × 3/1	64		
		Convolution Kernel	1 × 1/1	16		
		Separable Convolutional Layer	5 × 5/1	64		
		Max Pooling Layer	3 × 3/1	-		
		Convolution Kernel	1 × 1/1	64		

^1^ SVM: Support Vector Machine. ^2^ FC1: Fully Connected Layer. ^3^ GRU: The Gated Recurrent Unit. ^4^ WELM: Weight Extreme Learning Machine. ^5^ VGGNet: Convolutional Neural Network architecture, designed by the Visual Geometry Group at the University of Oxford. ^6^ QDNN: Deep Neural Networks with Quantum Layers. ^7^ Representing the four emotion categories.

**Table 10:** A quantitative comparison between proposed model and existing models.

Model	Dataset	Accuracy (per Dataset)	Precision	Recall	F1-score	ROC
Hybrid [11]	FER2013	84.4%	NA	NA	NA	NA
	CK+	95.1%				
	KDEF	98.5%				
	KMU-FED	98.6%				
AHTM [9]	KDEF	99.69%	98.86%	98.86%	98.86%	NA
	KMU-FED	99.50%	97.25%	97.54%	97.39%	
DFEER [10]	CK+	95.92%	93.57%	95.63%	94.60%	NA
	KMU-FED	94.73%	92.54%	94.42%	93.48%	
TLDFER-ADAS [18]	FER2013	99.31%	96.06%	96.71%	96.36%	98.15%
	CK+	99.29%	96.83%	93.32%	94.87%	96.27%
Proposed Model	Distraction	98.68%	95.8%	100%	97.8%	98.6%
	Drowsiness		100%	94.1%	96.9%	
	Neutrality		96.5%	97.6%	97.1%	
	Fear		100%	100%	100%	

**Table 11:** Max78000fthr microcontroller features.

Features	Details
Dual-Core Ultra-Low-Power Microcontroller	- Convolutional Neural Network Accelerator - 512 KB Flash Memory - 128 KB SRAM - 16 KB Cache - 12-Bit Parallel Camera Interface - MAX20303 Wearable PMIC with Fuel Gauge - Charge from USB - On-Board DAPLink Debug and Programming Interface for Arm Cortex-M4 processor with FPU - Breadboard Compatible Headers - Micro USB Connector - Micro SD Card Connector
Integrated Peripherals	- RGB Indicator LED- User Pushbutton - CMOS VGA Image Sensor - Low-Power Stereo Audio CODEC - Digital Microphone- SWD Debugger - Virtual UART Console - 10-Pin Cortex Debug Header for RISC-V Coprocessor

**Table 12:** Proposed model performance demonstration.

Model	Dataset	Accuracy	Precision	Recall	F1-Score	Average ROC
Proposed Model	Distraction	98.68%	95.8%	100%	97.8%	98.6%
	Drowsiness		100%	94.1%	96.9%	
	Neutrality		96.5%	97.6%	97.1%	
	Fear		100%	100%	100%	

**Table 13:** A comparison between proposed model and existing driver alert models.

System	Hardware Used	Processing Efficiency	Detection and Alert Methods	Latency
[59]	Raspberry Pi 3, Pi Camera, IoT Modules	Limited processing power, may struggle with real-time detection	Eye Aspect Ratio (EAR), facial landmarks, email alerts	Slower than dedicated AI chips
[60]	Camera, fingerprint sensor, robot arm	Multi-stage alert system adds complexity, slower processing	EAR, fingerprint verification, vibration alerts	Higher response time due to multi-stage alerts
[61]	Deep Learning on CPU/GPU	Higher processing needs, requires powerful hardware	Deep Learning Emotion Recognition (Facial Analysis)	Potential high latency
Our Model	MAX78000FTHR (AI-accelerated MCU)	Optimized for edge AI, low power consumption, real-time processing	AI-based sound detection, emotion analysis	Low-latency edge processing (Fast)

**Table 14:** A Comparison between proposed model and voice-based driver alert models.

System	Accuracy	Cost	Portability	Scalability	Power Consumption	Emotions Detected
[59]	97.7%	USD 150 Approx.	Many Components	Limited Scalability	Higher	Drowsiness
[60]	NA	USD 120 Approx.	Many Components	Complex: Fingerprint, Vibration, Robot Arm (Low Scalability)	Higher	Drowsiness
[61]	NA	USD 130 Approx.	Many Components	Limited Scalability	Low	Happy, Sad, Angry, Surprise, Disgust, Neutral and Fear
Our Model	98.6%	USD 75 Approx. (Affordable)	Portable	Highly Scalable	Low	Drowsiness, Distraction, Fear/Panic, and Neutrality

## Data Availability

The data presented in this study are available upon request from the M.A.A. author.
